# Updates on the Advantages and Disadvantages of Microscopic and Spectroscopic Characterization of Magnetotactic Bacteria for Biosensor Applications

**DOI:** 10.3390/bios15080472

**Published:** 2025-07-22

**Authors:** Natalia Lorela Paul, Catalin Ovidiu Popa, Rodica Elena Ionescu

**Affiliations:** 1 Materials Science and Engineering Department, Faculty of Materials and Environmental Engineering, Technical University of Cluj-Napoca, 400641 Cluj-Napoca, Romania; natalia.paul@utt.fr (N.L.P.); catalin.popa@stm.utcluj.ro (C.O.P.); 2 Light, Nanomaterials and Nanotechnologies (L2n) Laboratory, CNRS UMR 7076, University of Technology of Troyes, 12 Rue Marie Curie, CS 42060, CEDEX, 10004 Troyes, France; 3 Eut+ Institute for Nanomaterials & Nanotechnologies EUTINN, European University of Technology, European Union

**Keywords:** magnetotactic bacteria, magnetic nanoparticles, characterization methods, advanced microscopy techniques, spectroscopic investigations, biosensors

## Abstract

Magnetotactic bacteria (MTB), a unique group of Gram-negative prokaryotes, have the remarkable ability to biomineralize magnetic nanoparticles (MNPs) intracellularly, making them promising candidates for various biomedical applications such as biosensors, drug delivery, imaging contrast agents, and cancer-targeted therapies. To fully exploit the potential of MTB, a precise understanding of the structural, surface, and functional properties of these biologically produced nanoparticles is required. Given these concerns, this review provides a focused synthesis of the most widely used microscopic and spectroscopic methods applied in the characterization of MTB and their associated MNPs, covering the latest research from January 2022 to May 2025. Specifically, various optical microscopy techniques (e.g., transmission electron microscopy (TEM), scanning electron microscopy (SEM), and atomic force microscopy (AFM)) and spectroscopic approaches (e.g., localized surface plasmon resonance (LSPR), surface-enhanced Raman scattering (SERS), and X-ray photoelectron spectroscopy (XPS)) relevant to ultrasensitive MTB biosensor development are herein discussed and compared in term of their advantages and disadvantages. Overall, the novelty of this work lies in its clarity and structure, aiming to consolidate and simplify access to the most current and effective characterization techniques. Furthermore, several gaps in the characterization methods of MTB were identified, and new directions of methods that can be integrated into the study, analysis, and characterization of these bacteria are suggested in exhaustive manner. Finally, to the authors’ knowledge, this is the first comprehensive overview of characterization techniques that could serve as a practical resource for both younger and more experienced researchers seeking to optimize the use of MTB in the development of advanced biosensing systems and other biomedical tools.

## 1. Introduction

Shortly after their discovery in the late 1950s, magnetotactic bacteria were initially believed to be confined to sediments and aquatic environments with near-neutral pH values and temperatures within the typical range [1]. However, subsequent research has demonstrated that their distribution is, in fact, ubiquitous. These bacteria can be found in a wide variety of natural habitats, including aquatic environments, moist soils, lakes, and both freshwater and saline ecosystems, as well as in extreme environments [2,3]. Extensive studies have been conducted on their distribution across numerous locations, providing global coverage, including regions such as the Brazilian and Pacific lagoons, the China Sea, the North Sea, rivers and lakes across Asia, as well as in both European (Germany, France, Italy, Portugal, Spain, United Kingdom, Russia) and American continents [4].

**Figure 1:**
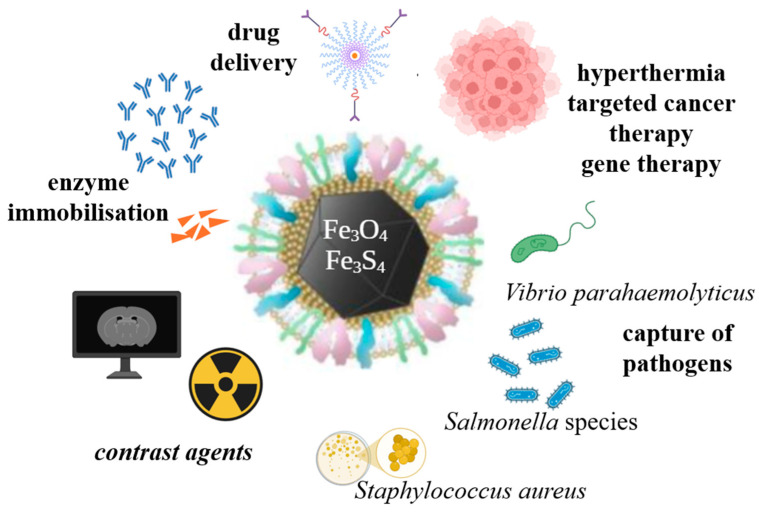
Schematic representation of magnetosome containing magnetic crystals (in the form of magnetite (Fe_3_O_4_) or greigite (Fe_3_S_4_)) and their promising applications in the biomedical field.

**Figure 2:**
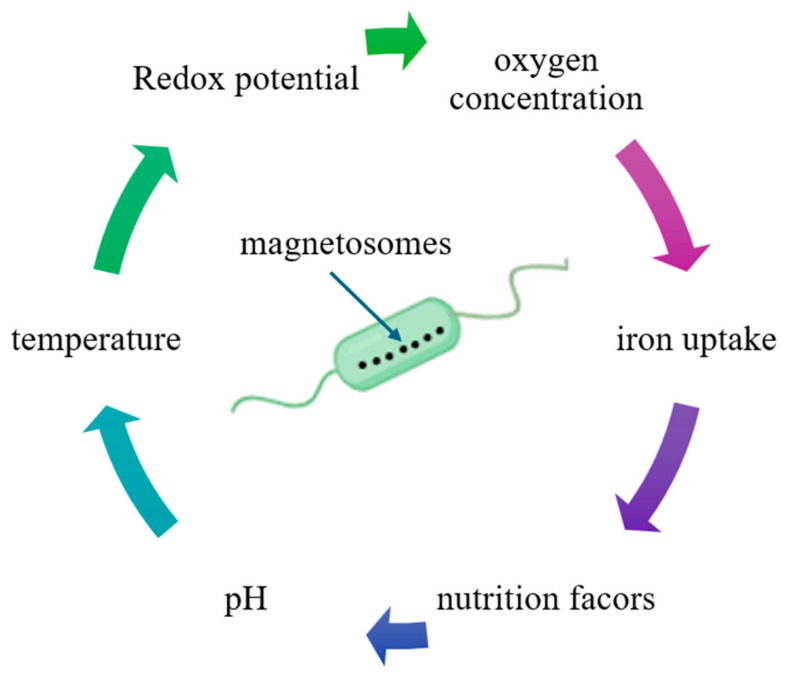
Factors contributing to the formation and development of magnetosomes.

MTB have garnered significant attention in recent years due to their unique characteristics, which distinguish them from other microorganisms, and particularly because of their diverse applications, especially in the medical and ecological domains. Their inherent magnetic properties place MTB at the forefront of preferences, surpassing other microorganisms and synthetic nanoparticles in various contexts. These bacteria are prokaryotic, Gram-negative organisms, exhibiting motility via flagella. They are capable of synthesizing intracellular magnetic crystals, with nanometric dimensions, composed of magnetite, iron oxides (Fe_3_O_4_), and/or greigite, iron sulfates (Fe_3_S_4_), which are surrounded by a lipid bilayer membrane (Figure 1). This membrane forms specialized organelles known as magnetosomes [3]. The formation and development of magnetosomes are strongly influenced by environmental conditions. Factors such as nutrient absorption, including iron and oxygen, carbon sources, as well as fluctuations in pH and temperature, directly impact the mineral composition synthesized by the bacteria and, consequently, the formation of magnetosomes (Figure 2) [5].

Magnetosomes are arranged in chain-like structures that function similarly to a compass, enabling the bacteria to navigate according to the Earth’s geomagnetic field [4]. This magnetic orientation facilitates the bacteria movement, allowing them to position themselves in environments conducive to their survival and growth, which is closely linked to optimal oxygen concentrations, redox gradients, and nutrient availability [2,3]. Thus, most MTB are microaerophilic, meaning they require low concentrations of oxygen, typically preferring an optimal oxygen concentration of up to 20 µM [6], with a natural habitat primarily found in the oxic–anoxic transition zone within chemically stratified aquatic environments, often located in the upper regions of sediment, at the bottom of the water column. Nonetheless, certain MTB strains are capable of thriving in extreme conditions, such as hot springs, alkaline saline lakes, or in deep aquatic environments [7,8].

On the other hand, the vertical component of the Earth’s magnetic field enables the MTB to align with the magnetic field lines, guiding them toward their preferred habitat [9], a phenomenon known as magnetotaxis. This capability enables the bacteria to navigate autonomously, without reliance on an external energy source [10]. For instance, Rismani Yazdi et al. (2018) [10] demonstrated, using the *Magnetospirillum magneticum* strain (AMB-1), that magnetotaxis enables MTB to orient themselves both perpendicular to the flow and in the opposite direction. The bacteria movement is influenced by the flow, the magnetic field, and their relative orientation. These findings are particularly relevant in the context of aquatic ecosystems, where MTB must often travel long distances to reach the oxic–anoxic transition zones. Through their research, the authors highlighted the potential use of these microorganisms in biomedical applications, such as targeted drug delivery and in vivo microrobotics for the precise targeting of specific cells or tissues [10].

MTB exhibit highly specific requirements for cultivation under laboratory conditions, demanding precise control over temperature, oxygen concentration, and nutrient supply in the culture medium. Oxygen concentration is crucial for the proliferation of MTB, while iron concentration plays a pivotal role in the formation of magnetosomes. Additionally, carbon concentration stimulates magnetosome formation and serves as an energy source in the culture medium. Temperature is another critical factor influencing the activity and growth of these bacteria [5]. Furthermore, the considerable diversity of MTB strains suggests that different strains may have distinct nutrient and chemical gradient requirements for optimal growth and division [11].

**Figure 3:**
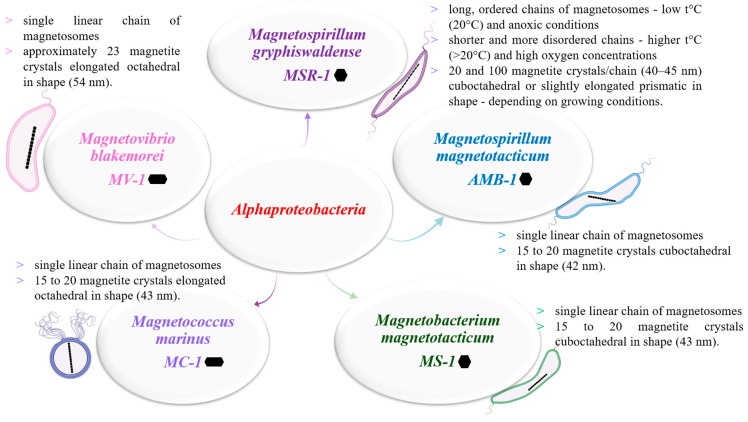
Schematic illustrations of the bacterial shapes of the main types of magnetotactic bacteria studied, belonging to the phylum *Alphaproteobacteria*.

In recent years, over 16 new MTB species have been discovered in mountain environments [12,13]. However, cultivating these species under laboratory conditions remains a significant challenge that requires further investigation. In contrast, species of magnetotactic bacteria that have been studied extensively, with well-established cultivation protocols, belong to the genus *Magnetospirillum* (class *Alphaproteobacteria*, phylum *Proteobacteria*), including strains such as *Magnetospirillum magneticum* (AMB-1), *M. magnetotacticum* (MS-1), and *M. gryphiswaldense* (MSR-1). In this regard, the species found in Figure 3 are those that have received the most concentrated attention from the scientific community both in terms of establishing cultivation protocols, respectively, identifying the conditions conducive to their growth in laboratory conditions, as well as in terms of the quality of the information obtained from their analysis. From the steps undertaken and from the research carried out, information was mainly obtained regarding the shape of the crystals contained by these species, the number of magnetosomal chains, as well as the size of the magnetic crystals contained [11].

Despite substantial progress in exploring and discovering new MTB strains, as well as recognizing their promising potential, several challenges remain. One such challenge pertains to the individual characterization of the bacteria and their magnetosomes, specifically the methods used for characterization via microscopy tools. In this context, this review aims to provide a comprehensive overview of the key aspects studied to date, including characterization techniques, the MTB strains employed, the advantages of these methods, and any limitations or drawbacks that complicate the evaluation process. A thorough understanding of the properties of MTB, as well as the magnetosomes and nanoparticles biomineralized by them, is crucial for advancing their application, particularly in the development and implementation of novel biomedical applications.

## 2. Promising Characteristics of Magnetotactic Bacteria in Biomedical Research

Magnetotactic bacteria are attracting increasing interest in biomedical research due to their unique ability to biosynthesize magnetosomes, magnetic nanocrystals, arranged in order in intracellular chains and enveloped in a lipidic-protein membrane. These magnetosomes exhibit a precise crystalline structure, uniform dimensions and a purity superior to those obtained by conventional synthetic methods. In addition, the biological membrane that surrounds them allows for efficient functionalization with bioactive molecules, an essential aspect in therapeutic and diagnostic applications. One of the most valuable characteristics of MTB is the high biocompatibility of magnetosomes, correlated with low toxicity and a promising and efficient degradation in biological systems. These qualities offer a major advantage compared to synthetic magnetic nanoparticles, which frequently raise problems of aggregation, stability, and safety in use. Thus, magnetosomes represent a natural, sustainable, and functional alternative to synthetic magnetic materials currently used in nanomedicine [14,15]. Due to their well-defined magnetic properties and the possibility of biological control over their synthesis, MTB and their magnetosomes are being intensively explored in applications such as magnetic resonance imaging (MRI), magnetically induced hyperthermia, targeted drug delivery, and gene therapies. In addition, the ability to move under the influence of a magnetic field allows them to be integrated into active transport systems or steerable nanodevices, opening new perspectives in localized therapies and in the precise diagnosis of pathologies of biomedical interest [14,15,16,17].

### 2.1. Magnetosomes Formation in MTB

The formation of the magnetosome chain is a multi-step process that exhibits slight variations depending on the species. For instance, in the *Magnetospirillum* species, cytoplasmic membrane invagination occurs first, followed by the precipitation of magnetic crystals made of greigite [18]. It is hypothesized that a sorting mechanism for magnetosomal membrane proteins may exist during membrane invagination and vesicle formation. In *Magnetospirillum magneticum*, different stages of crystal precipitation have been observed in cells grown under iron-limited conditions, where empty magnetosome vesicles are arranged in chains and attached to the membrane. Additionally, a small percentage (35%) of magnetosomes exhibited membrane invaginations, suggesting that in other cases, these invaginations break off and form detached membrane vesicles [19].

A subsequent step in magnetosome chain formation is the internalization of iron into the magnetosomal vesicles. Studies on *Magnetospirillum gryphiswaldense* have demonstrated a mechanism in which iron, essential for the biomineralization of magnetite crystals, is transported through the cytoplasmic membrane directly to the magnetosome membrane, bypassing the cytoplasm. This finding indicates that iron uptake, assimilation/internalization, and storage, as well as the formation of magnetic crystals, are distinct stages in the process [20,21]. Once iron is internalized into the magnetosome vesicle, nucleation and maturation of the magnetic crystals begin. A key point is that iron absorption occurs rapidly and influences the morphology of the magnetic crystals within the magnetosomes [22,23].

In all magnetotactic bacteria, magnetosomes are organized into single or multiple chains that are aligned in a manner similar to actin filaments [22]. The size and shape of magnetosomes, as well as the nanoparticles within them, are of great significance in the characterization of MTB and in understanding the mechanisms that underlie their principal functions. These characteristics are crucial for elucidating the bacteria’s potential applications across various sectors.

### 2.2. Biomineralization of Magnetosomes in MTB

Studying the properties and biomineralization mechanisms of MTB necessitates their isolation from natural environments, followed by their cultivation and genetic manipulation in the laboratory. The first strain cultivated, *M. magnetotacticum* MS-1, has facilitated numerous molecular and structural characterizations (approximately 30 to date) [22]. However, challenges in cultivating colonies of MS-1 on agar medium prompted has increased the focus on other strains that could support further studies. Agar is a semi-solid growth medium for isolating unknown MTB or for growing different species that are sensitive to oxygen and redox gradients [11]. *M. gryphiswaldense* MSR-1 and *M. magneticum* AMB-1 have proven easier to cultivate than MS-1, making them model organisms for studying magnetotactic bacteria. These strains have been studied using both cell and molecular biological techniques, as well as genetic approaches to investigate the roles of specific proteins and enzymes in the formation of magnetic crystals and magnetosomes [22] (Figure 3).

**Figure 4:**
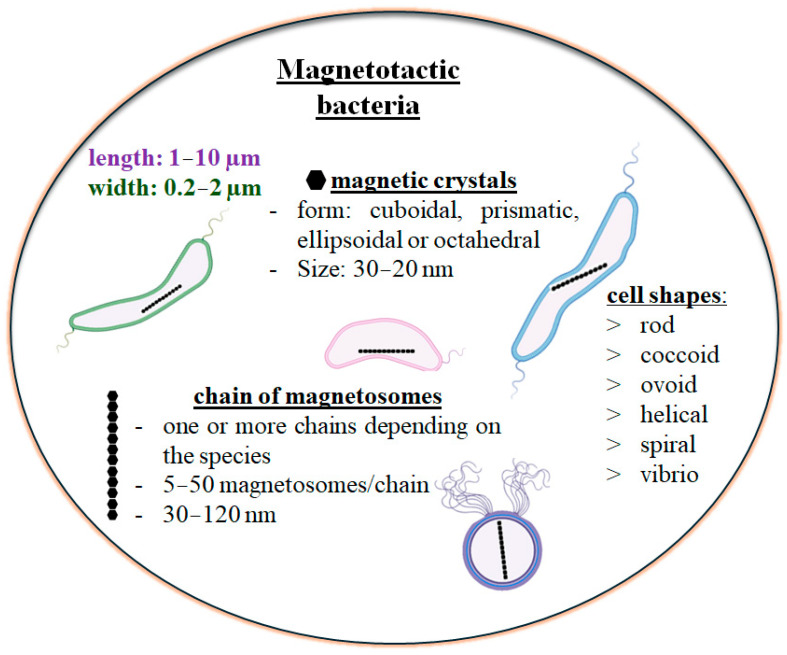
Shapes of magnetotactic bacteria and their magnetosomes.

Magnetotactic bacteria possess the ability to regulate intracellular mineralization, as evidenced by their capacity to form magnetic particles of greigite and/or magnetite. Beyond mineralizing these particles, the magnetosomes in MTB enable the bacteria to align with and navigate along the Earth’s magnetic field lines. Additionally, magnetosomes exhibit enzyme-like activity (peroxidase, catalase, oxidase-like activity), contributing to the removal of reactive oxygen species. MTB also enhance stress tolerance by incorporating metals such as Zn, Co, Cu, and Mn into the magnetosomes, and play a role in the biogeochemical cycling of carbon, nitrogen, sulfur, potassium, and iron [22,23,24]. The shape, composition (5–50 magnetic crystals per cell) [25], and arrangement of magnetosomes vary among species (15–30 magnetosomes per bacterium) [26], and similarly, the dimensions of the magnetic crystals differ, ranging from 30 to 120 nm [5,22,24] (Figure 4).

## 3. Data Collection

**Figure 5:**
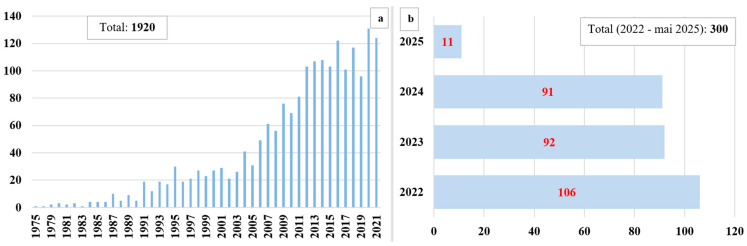
Schematic representation of the number of publications registered in the scientific database, Web of Science (1920), since the discovery of magnetotactic bacteria (**a**) and in the last 40 months (January 2022–May 2025) (**b**).

To obtain a comprehensive understanding of the current state of knowledge regarding magnetotactic bacteria, a thorough literature review was conducted. The review began with a search of the Web of Science database, one of the most updated and extensive sources for discovering new species of magnetotactic bacteria, cultivation methods, and protocols, as well as approaches for evaluating, characterizing, and describing both the bacteria and the nanoparticles biomineralized by them. The preliminary search utilized the key term “magnetotactic bacteria”, which yielded 2220 publications. A preliminary analysis of these data (Figure 5) revealed a noticeable upward trend in the interest surrounding magnetotactic bacteria since their discovery. Notably, in the past two decades, there has been an exponential increase in the number of published articles. This trend strongly reflects the significant interest in exploring the unique properties of these bacteria, understanding their mechanisms of action, optimizing cultivation conditions, and investigating their potential applications across various sectors.

## 4. Data Analysis

The search was further refined by filtering for articles published within the last three years, specifically from 2022 to 2024, and by including review articles, open-access documents, and publications in English. This refinement resulted in 289 articles. To narrow the results to our area of interest, we applied a new combination of key terms: “magnetotactic bacteria” AND “magnetic nanoparticles” AND “biomineralization” AND “optical methods” AND “atomic force microscopy” AND “scanning electron microscopy” AND “transmission electron microscopy” AND “spectroscopy techniques”. This additional refinement generated 59 articles, which were then selected for more detailed analysis.

**Figure 6:**
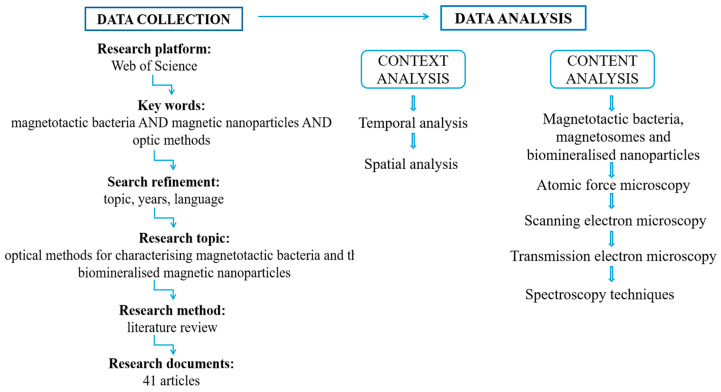
Schematic representation of the methodology of research.

Following a systematic review of the selected publications concerning the methods for analyzing, characterizing, and evaluating magnetotactic bacteria and their biomineralized nanoparticles, the gathered information was organized based on both context and content analysis parameters (Figure 6). The context analysis was performed by examining the spatial and temporal aspects, with the aim of highlighting the ongoing and consistent research focused on the applicability of magnetotactic bacteria. Meanwhile, the content analysis was structured around the central research topic and the emerging variables: the characteristics of magnetosomes and magnetic nanoparticles biomineralized by magnetotactic bacteria, and the optical and microscopy techniques employed for their evaluation, analysis, and characterization.

### 4.1. Context Analysis

Research focused on the methods for characterizing various strains of magnetotactic bacteria has contributed to the development of a comprehensive understanding of this subject. The publications identified offer a thorough analysis of the current research landscape, particularly highlighting the key challenges faced by researchers in isolating, cultivating, and characterizing different MTB strains.

**Figure 7:**
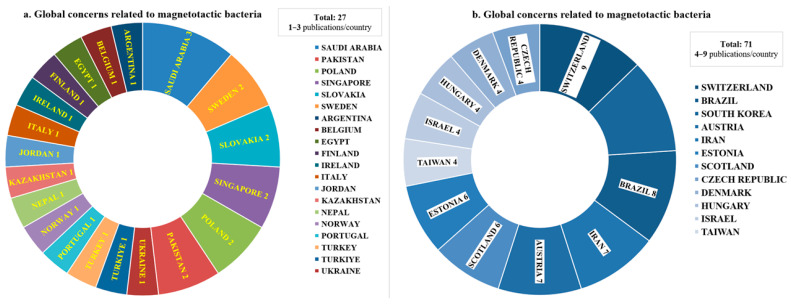

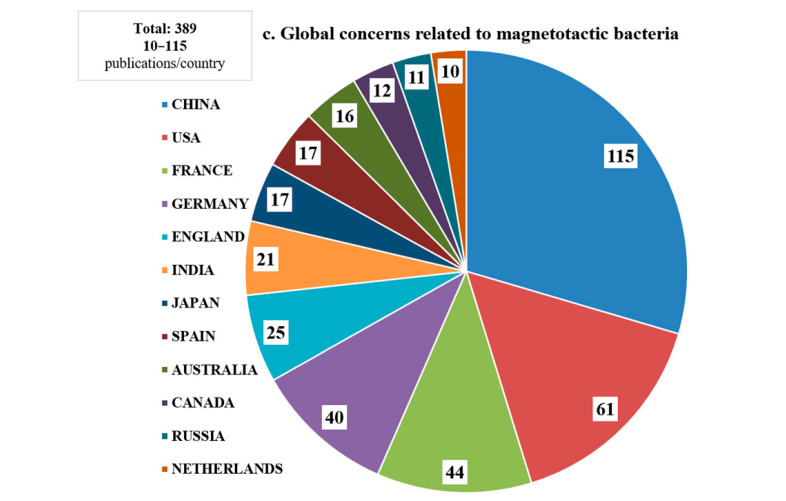
Graphic representation of the global concerns related to magnetotactic bacteria over the years 2022, 2023, 2024, and (May) 2025, according to the Web of Science database. Representation of number of publications per country: 1–3 publications (**a**), 4–9 publications (**b**), respectively, and 10–115 publications (**c**).

The context analysis was conducted based on the temporal and spatial criteria of the identified sources. As shown in Figure 5b, during the years 2022, 2023, 2024, and until May 2025, interest and research related to these bacteria have remained consistently high, reflecting both the ongoing progress in the field and the determination to fully explore the potential applications of magnetotactic bacteria. Additionally, Figure 7 illustrates the global distribution of research published in recent years, which is indicative not only of the widespread interest in the subject but also of the ubiquitous distribution of magnetotactic bacteria. This is further demonstrated by the identification and characterization of new bacterial strains during the period from 2022 to May 2025.

### 4.2. Content Analysis

Since the discovery of magnetotactic bacteria and their unique ability to biomineralize magnetic nanoparticles within their structure, significant efforts have been made to understand their mechanisms of action, the structure and formation of magnetosomes and nanoparticles, the characterization of biomineralized MNPs, as well as the methods for collecting, isolating, and cultivating MTB. Research has also focused on exploring the advantages of their use and their potential applications.

Our interest in investigating the methods for analyzing MTB arises from their unique properties and the need to identify effective, efficient, and insightful methods that can help the scientific community uncover the advantages and potential limitations of utilizing these bacteria. Additionally, these methods aim to identify strategies for overcoming any associated challenges.

Although the application potential of MTB has been widely demonstrated across various fields and sectors, their cultivation under laboratory conditions remains a key focus of research. The strains that exhibit the best adaptation to laboratory conditions, with well-documented survival and established cultivation protocols, are *Magnetospirillum magneticum* (AMB-1), *Magnetobacterium magnetotacticum* (MS-1), and *Magnetocspirillum gryphiswaldense* (MSR-1) [11,22,27].

## 5. Microscopic Characterization of MTB

**Figure 8:**
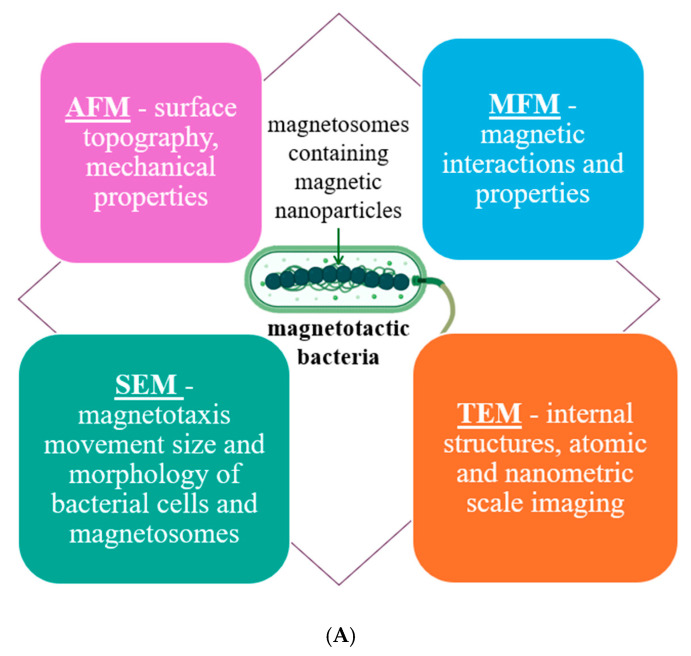

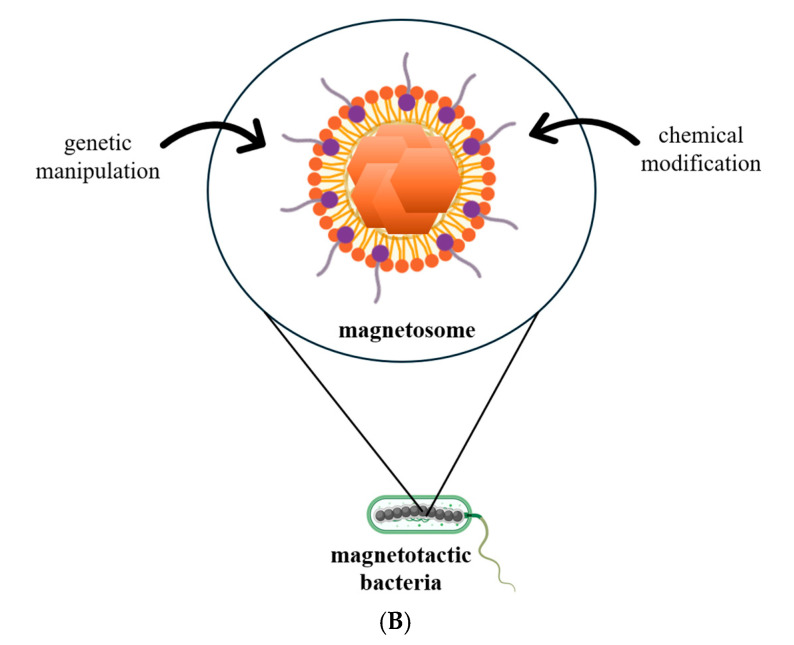
Main characteristics of MTB evaluated using microscopy techniques (**A**) and the engineering pathways for magnetotactic bacteria and their magnetosomes (**B**).

Observation of magnetotactic bacteria using conventional optical microscopy, including phase contrast and differential interference contrast (DIC), allows for rapid and non-invasive imaging of the general morphology and motility of MTB microorganisms. Unlike phase contrast, which is based on total refractive index, *DIC* is based on the local change in refractive index and more suitable for thicker samples than phase contrast. Also, DIC facilitates the identification of live unstained cells with no phase ring in the objective and a larger depth of field. However, in DIC images, the cell outline is much more difficult to extract, being made of both light and dark regions and making it more difficult to distinguish intracellular structures (e.g., the detailed observation of magnetosomes or their precise organization within the cell). Furthermore, these methods do not provide information on the chemical composition or magnetic properties of the observed structures, limiting the understanding of the functional mechanisms of MTB. For all these reasons, the advanced characterization of bacteria requires the addition of high-resolution techniques. In order to ensure a comprehensive and varied assessment, a wide range of optical microscopy techniques are employed to morphologically characterize nanomaterials, specifically magnetosomes. Among these, the most frequently used and suitable methods of MTB surface characterization are scanning electron microscopy (SEM) using dehydrated cells, while transmission electron microscopy (TEM), atomic force microscopy (AFM), and magnetic force microscopy (MFM) work with live specimens *(*Figure 8A).

While other techniques may also be utilized (metagenomics, single-cell genomics, or CRISPR-Cas9 and genetic engineering) (Figure 8B), this review focuses primarily on those methods that help describe the morphology, shape, and size of the nanoparticles contained within these bacteria, as these aspects are crucial in defining the performance of magnetotactic bacteria in various applications.

Nanoparticles are substances with at least one dimension on the nanometric scale, where the surface-to-volume ratio significantly increases. This characteristic is particularly relevant in the context of their applications due to their exceptional physical, chemical, magnetic, optical, and electrical properties. Their performance, whether topographic or tomographic, is entirely dependent on their morphology. High-resolution microscopes are required to assess these properties. For instance, scanning electron microscopy (SEM) provides detailed information about the surface texture of nanomaterials and nanoparticles [28]. Transmission electron microscopy (TEM) offers insights into the shape and size of nanoparticles, while atomic force microscopy (AFM) is utilized to assess the surface roughness of magnetotactic bacteria, as well as the surface forces, size, and thickness of nanomaterials [29].

Each of these methods has its advantages and limitations. In the following section, we will briefly outline each technique, discussing its strengths, weaknesses, and the type of information it provides regarding magnetotactic bacteria.

***Techniques for characterizing the internal structure of bacteria***

### 5.1. Transmission Electron Microscopy in the Analysis of MTB

Transmission electron microscopy is a high-resolution technique that uses beams of electrons. These electrons are transmitted through samples and allow detailed images to be obtained at the nanometer or atomic scale. The electron beams are accelerated to high voltages, between 100 and 300 kV, thus ensuring a resolution of the nanometer order [30].

This microscopy technique is extremely versatile, providing a wide range of information about the samples analyzed. It makes it possible to obtain information at an atomic resolution about the internal structure of materials, such as the morphology, shape, size, or distribution of particles; orientation and arrangement of atoms; as well as information that makes it possible to distinguish between crystalline and amorphous materials. Coupling TEM with an X-ray detector allows the analysis and chemical composition of samples through elemental analysis and mapping [31].

The precision, accuracy, and versatility of this technique have allowed TEM to find its utility in numerous fields and branches of science, such as materials science, nanotechnology and nanomaterials analysis, chemistry, electronics, forensic geology and forensic analysis, and, last but not least, in biology and medicine [31,32,33]. Regarding the latter, TEM plays a crucial role due to its ability to provide detailed information about the internal structure of cells and molecules. Using the electron beam transmitted through the ultrafast sample, TEM allows obtaining images at a resolution of the order of nanometers or even atoms [31,34].

In general, biological samples are fixed, dehydrated, and embedded in an epoxy resin, and then sectioned using an ultramicrotonne. In the case of fragile biological samples, the cryo-fixation technique is used for their structural preservation and subsequent analysis [31,35].

Magnetotactic bacteria are, so far, the only known group of prokaryotes that have both biomineralization and magnetoreception capacity. For this reason, numerous studies have been directed toward their characterization, the analysis of magnetosomes, and the observation of the phenomenon of magnetotaxis.

One of the first microscopy techniques that was used to analyze and characterize these bacteria was transmission electron microscopy [36,37]. This provided essential information about MTB; among the first observations, it was possible to identify magnetosomes and intracellular particles of magnetite and greigite, biomineralized by them. The presence of the flagellum was also highlighted and the analysis of the magnetotactic behavior of magnetotactic strains was analyzed, thus making it possible to demonstrate their orientation in the magnetic field, as well as to explain their behavior in dependence on the oxygen concentration [37]. Since then, numerous strains of magnetotactic bacteria have been analyzed and characterized in order to understand their mechanism of operation and possible applications in various fields of activity, but especially in the biomedical sphere.

As an illustrative example, we take the study carried out by Zhang et al. (2022) [38]. They proposed the analysis of anhysteretic remanent magnetization (ARM) in order to quantify the concentration of ferrimagnetic particles in rocks and sediments. Anhysteretic remanent magnetization is a parameter used to evaluate information related to variations in the Earth’s magnetic field and environmental changes. To this end, they analyzed marine sediments from the Eastern Pacific Ocean, the Antarctic margin, the Arctic Ocean, and the South China Sea [38].

Magnetic extracts from the sampled sediments were analyzed by transmission electron microscopy and revealed the presence of magnetofoscillates with high iron and oxygen content. These were in close agreement with the presence of magnetite, a fact validated by additional investigations carried out by lithostratigraphy and water geochemistry [38].

In their study, Kadam et al. (2024) [39] reported the discovery of magnetofossils extracted from the Bay of Bengal. These are, according to the reports and investigations of these researchers, the youngest giant magnetofossils reported to date. Analysis and characterization using scanning electron microscopy allowed the identification of the shape of these magnetofossils, and transmission electron microscopy confirmed their morphological variability, as well as their magnetic component [39].

Similarly, Zhang and Wang (2024) used TEM to directly confirm the presence of magnetofossils in sediments, and analyses coupled with EDS allowed the determination of their iron and oxygen composition, suggestive of magnetite composition [40].

Masó-Martínez et al. (2023) [41] performed a comparative analysis of disruption techniques (enzymatic treatment, probe sonication, and high-pressure homogenization) to study their effect on the length of the magnetosome chain of the strain *Magnetospirillum gryphiswaldense* MSR-1, as well as on the integrity and aggregation state of the isolated magnetosomes. The analyses were performed using TEM, JEM-2100 F (JEOL, Herts, UK) at 200 kV and equipped with a Gatan Orius CCD camera (Pleasanton, CA, USA), and the dynamic light scattering technique, coupled with nano-flow cytometry. The TEM results obtained showed that high-pressure homogenization allowed optimal preservation of the integrity of the magnetosome chain, while enzymatic treatment generated its cleavage. The purpose of the experiment conducted was based on examining the effect of these types of cell destruction treatments on the integrity of the magnetosome chain in order to study and identify the potential of these magnetosomes to lay the foundations for a new generation of functional materials [41].

An interesting perspective in the study of the strain *Magnetospirillum magneticum* AMB-1 was brought by Gandia et al. (2023) [42], who cultivated the MTB strain in three different culture media to analyze how they influence the structural, morphological, and magnetic characteristics of the magnetosome chains. The culture media they used were Wolfe mineral solution medium, growth medium without Wolfe mineral solution, and a standard medium for reference [42].

The investigated characteristics were analyzed by transmission electron microscopy (JEOL JEM-1400 Plus at 120 kV), X-ray absorption spectroscopy, and X-ray circular magnetic dichroism (ALICE station at the PM3 beamline of BESSY II in Berlin, Germany). The TEM characterizations allowed the researchers to observe and compare the bacteria grown in different culture media conditions. Thus, they observed that in all three media, the bacteria developed properly, and they presented the spirilla shape and fragmented chains specific to this strain. Moreover, the number of magnetosome subchains was comparable, and the average sizes were also similar. The significant difference, however, was observed in the size of the magnetosomes, which is representative of their magnetic response [42].

Therefore, regardless of the medium used, the AMB-1 bacteria were capable of growth and development, forming complete chains of magnetosomes, but the magnetic response was different. The evaluation of their magnetic response was evaluated by measuring the heating efficiency within the hyperthermia processes to which they were subjected. Bacteria grown on Wolfe mineral medium (MSGM+W) had a pronounced increase in magnetocrystalline anisotropy and magnetic coercivity. In contrast, bacteria grown in the medium without minerals (MSGM-W) showed high uniaxial anisotropy with increased heating efficiency. Even in the absence of minerals, this medium presents itself as a promising alternative for bacterial cultivation [42]. These results indicated and emphasized the importance of the culture medium in controlling and regulating the magnetic response of these bacteria. This aspect can be further exploited for cultivation in preferential culture media, adapted for particular medical applications in which magnetotactic bacteria are desired [42].

TEM is therefore an indispensable tool in the characterization of magnetotactic bacteria and is among the main method for analyzing the shape, size, and arrangements of magnetosomes, as well as mineralized magnetotic crystals [25,32,43,44,45,46]. In Table 1, the main advantages and disadvantages of using TEM in MTB characterization are summarized, and the main bacterial strains analyzed by TEM are summarized in the table below (Table 2).

***Techniques for characterizing bacterial surfaces***

### 5.2. Scanning Electron Microscopy in the Analysis of MTB

SEM is a microscopy technique that allows the detailed and high-resolution analysis of the surface of the analyzed samples, being used in numerous fields and sectors of activity. The scanning electron microscope is, from a functional point of view, similar to optical instruments, except that it uses a focused beam of electrons instead of a light source. The beam, generated by an electron gun, is focused on the surface of the sample and, in this way, allows obtaining information about the structure and composition of the analyzed specimen [49]. Directed through metal apertures and specialized lenses, the emitted electrons interact with the atoms inside the sample under analysis and determines the obtaining of several types of signals, including secondary electrons, backscattered electrons, and characteristic X-rays. The signals obtained in this way are, in fact, information regarding the topography, surface, composition, and properties of the specimen [50].

Unlike other optical microscopy instruments, SEM operates in a vacuum, which requires a stable power source, proper cooling system, and use in a vibration-free space sheltered from ambient magnetic and electric fields. The operating principle of the SEM relies on the generation of a beam from the electron gun, descending into a column and onto a series of electromagnetic lenses. The latter are called solenoids and are actually tubes wrapped in coils. They are adjusted so as to allow the appropriate focusing of the incident beam on the sample under investigation. The interaction with the sample is given by the acceleration speed of the incident electrons before being focused and directed onto the specimen. At the moment of interaction, the electrons are released from the surface of the sample, and their scattering patterns are those that generate information about shape, size, and composition [51].

The signals generated by the interaction with the sample are of several types. Secondary electrons are emitted by the surface of the analyzed sample as a result of the collision with the electrons in the primary beam; they are responsible for information on the topography of the surface of the analyzed samples and provide details on the structure, texture, and morphology of the surface. Backscattered electrons, as the name suggests, are those electrons reflected back from the sample. They are sensitive to the atomic density of the material and provide details on the chemical composition of the sample. The detectors, used to capture the scattered electrons, are part of a more varied and diversified palette. They can also be adapted to capture secondary electrons, as well as X-rays [52,53]. Practically, X-ray dispersive spectroscopy is a complementary technique that can be used in conjunction with SEM to analyze the chemical composition of materials or samples. When electrons in the primary beam strike atoms in the sample, they can release specific X-rays, the measurement of which allows the determination of the chemical element present in the analyzed material [54].

A distinctive and characteristic aspect is given by the fact that the electron beam steering, the interaction with the sample, and the acquisition of images in SEM take place in a vacuum. For this reason, the specimens subjected to analysis require prior preparation so that they can be introduced into the vacuum chamber. Preliminary preparation of the samples is necessary so that they can be subjected to the low pressure found inside the vacuum chamber. In the case of biological samples, for example, one of the preliminary preparations involves dehydrating the specimens and immobilizing them on a support. In addition, in the case of most biological samples, they also need to be coated by spraying with various substances/metals. The latter aspect is a consequence of the fact that biological samples are non-conductive samples [55].

A highly suggestive example in this context is the study carried out by Bektas and Yildirim (2025) [56] to analyze the structure of bacterial cellulose (BC) produced by *Bacillus* strains. Bacterial cellulose is a natural polymer with potential in numerous applications in various industries. They analyzed several strains that produce bacterial cellulose, including *Bacillus amyloliquefaciens* B1, *Bacillus subtilis* B2, *Bacillus velezensis* B3, *Bacillus siamensis* B4, and *Bacillus amyloliquefaciens* B5 [56]. In this regard, they used a high-vacuum scanning electron microscope, JEOL JSM-6360LV, at a magnification of 20,000. The bacterial samples used were dried and coated with gold and were analyzed at 20.0 kV. SEM analysis revealed the physical structure of BC, characterized by a heterogeneous network of interconnected fibers. This network confers a high water retention capacity and, consequently, increased humidity maintenance. Its evaluation by SEM revealed a complex structure and an intricate arrangement of the fibers that were grouped in the form of dense ribbon bundles with variable thicknesses, depending on the bacterial strain (28–108 nm) [56].

Another example, but this time one targeting magnetotactic bacteria, revealed the usefulness of SEM in the characterization of extremophilic magnetotactic bacteria. Oestreicher et al. (2022) [57] identified the presence of MTB in hot and moderately thermophilic hot springs (Mickey Hot Springs) rich in arsenic (1 mg/L). They investigated the possibility of the existence of these bacteria in different locations and habitats with extreme conditions, such as high temperature, high pH, or the presence of toxic substances in the environment [57].

The identified MTB belong phylogenetically to the *Nitrospirae* phylum and were analyzed and characterized using several microscopy techniques. Oestreicher et al. (2022) [57] used TEM for morphological analysis of the cells, respectively, to study the size and shape of the magnetosomes. On the other hand, to determine the chemical composition of the magnetosomes, they used scanning transmission electron microscopy (STEM) coupled with energy dispersive spectroscopy (EDX). Unlike SEM, which uses reflected electrons to generate information about the surface of the analyzed material, STEM uses a combined approach of microscopy techniques to provide information about both the surface of the material and its internal structure. Coupling with an X-ray detector enriches the quality of the information obtained by providing details about the elemental chemical composition of the materials [54].

The analyzed MTBs were placed on a carbon-coated copper grid and Formvar (Ted Pella, Redding, CA, USA) and subsequently analyzed using an FEI Tecnai F20 STEM (FEI Company, Hillsboro, OR, USA)at an accelerating voltage of 200 keV; for the analysis of magnetic crystals, they used an EDX F20 energy-dispersive X-ray spectrometer (EDAX, Mahwah, NJ, USA) [57].

Through this research, the authors found that the cells had a single polar flagellum, and the chain of magnetosomes inside the cell was unique, while the magnetic crystals (40 to 136 nm) were bullet-shaped and composed of iron and oxygen [57]. Another aspect highlighted in this study is that MTB removes arsenic from solution. However, the data collected and analyzed on bacterial magnetosomes did not reveal arsenic content in the magnetite crystals, but it was identified in the bacterial cell. This aspect suggests that the arsenic-containing environment does not affect the synthesis of magnetic crystals by these bacteria. The ability of MTB to tolerate high concentrations of arsenic can be further used in the creation of biosensors for the detection of this substance in contaminated waters or even for use as metalloid biosensors for public health programs [57].

HR-SEM is an advanced scanning microscopy technique that allows for more detailed images of the analyzed specimens. It differs from SEM in that it uses a much more focused and precise electron beam that allows for higher resolution images [50].

The use of HR-SEM—FEI Quanta 250 FEG, at a voltage of 10 kV in a high vacuum, allowed the characterization and assessment of the size and morphology of bacterial cells, as well as magnetosome chains [29].

Marcano et al. (2022) [47] attempted a method of acquiring experimental data, in close agreement with theoretical modeling, in order to obtain quantitative information about the magnetic anisotropy of magnetosomes. The latter aspect refers to both the magnetic anisotropy constant and the direction of the magnetic axis of magnetosomes. The method they used was based on the use of magnetic imaging techniques with nanometer-scale resolution. In this sense, they tested this hybrid approach on the magnetotactic bacterial strain *M. blakemorei* MV-1, which they analyzed by scanning transmission X-ray microscopy (STXM) and TEM [47]. Compared to SEM, STXM uses a soft X-ray beam that passes through the sample, and the image is formed based on the X-rays absorbed in different regions of the sample [50].

Therefore, Marcano et al. (2022) used a hybrid method combining both experimental and theoretical approaches to specifically explore the magnetic properties of magnetotactic bacteria [47].

Another revealing example of the utility of microscopy systems was revealed by the study conducted by Schaible et al. (2022) [58]. They analyzed an unidentified species of magnetotactic bacteria using a combination of microscopy techniques to analyze and reveal their characteristics.

The bacteria were collected from a tidal pool in the USA (Falmouth, MA, USA). The techniques used in the analysis of bacteria are from a broader and more varied spectrum, including fluorescence in situ hybridization (FISH), scanning electron microscopy (Zeiss Microscopy GmbH, Jena, Germany), confocal RAMAN microscopy (Horiba Jobin-Yvon, Kyoto, Japan), and nanoscale secondary ion mass spectrometry (NanoSIMS) (CAMECA, Gennevilliers, France) [58].

Through them, Schaible et al. (2022) demonstrated the usefulness of the combined use of several microscopy techniques so that it is possible to obtain and adapt the workflow at the cell level for the investigation of information related to morphology, physiology, or taxonomy [58].

In Table 3, we have summarized the main MTB strains characterized using SEM.

For a complete picture of the utility of SEM in bacterial characterization, we refer to Table 4 for the main advantages and disadvantages of this technique.

### 5.3. Atomic Force Microscopy in the Analysis of MTB

Since its discovery, atomic force microscopy has been employed to analyze a wide range of materials, including soft matter, facilitating the evaluation of their properties and physicochemical characteristics. AFM is a high-resolution technique that allows the visualization, manipulation, and characterization of molecular and atomic surfaces of materials, nanoparticles, and even various biological structures [60]. This instrument is versatile in its capability to approach and analyze various types of surfaces [61].

The AFM system consists of a nano-positioning system, an electronic controller, and a flexible cantilever that ends in a sharp tip, which detects external forces during movement [62]. As the tip physically scans the surface of the sample, it generates topographical measurements. The information provided by AFM includes data on roughness, surface topography, and the mechanical properties of the sample being analyzed [60,63,64].

AFM has proven useful across numerous sectors, including biology, medicine, physics, and materials science and engineering [65]. The analysis of biological samples, in particular, holds significant importance for understanding fundamental biological principles, identifying operational modes, and examining the complex interactions involved. AFM meets the need for a microscopy technique that offers multiple ways to observe dynamic biological processes, providing both spatial and temporal resolution while offering versatile imaging options, as shown in Table 5. This versatility is especially valuable in biomedical research, where understanding cellular structures and their interactions, such as those with bacteria, is crucial. AFM enables the assessment of mechanical properties, surface interactions, and forces at high resolution, which are vital for studying these processes in the human body and other biological systems [60,66].

In AFM, interaction with the sample occurs through a flexible probe equipped with a tip, actuators, and sensors. Probes are typically made from materials such as silicon dioxide, silicon nitride, or other silicon-based substances. The nano-positioning system adjusts based on the operating mode, deformation characteristics, and oscillation properties of the cantilever, enabling fine control over the relative distance between the probe tip and the sample surface [70]. Ensuring high performance in the imaging system and avoiding damage to biological samples are critical requirements for AFM systems.

The versatility of AFM, including its ability to operate in vacuum, ambient, or liquid media, makes it a preferred method for analyzing biological samples in a range of environments [71]. AFM offers several analysis modes, with each tailored to specific sample types and the information desired. These modes include contact mode, non-contact mode, and tapping/intermittent mode [66,70].

*In contact mode*, the cantilever tip directly interacts with the surface of the sample, providing detailed information about the structure and topography of the sample. As the tip continuously contacts the sample during the analysis, the deflection of the cantilever is recorded by a photodetector, which generates an electrical signal proportional to the deflection or tilt of the tip [66,70].

*In non-contact mode*, as the name suggests, the cantilever tip does not physically touch the sample. Instead, it oscillates just above the sample’s surface (usually at a distance of up to 150 Å). This mode detects the surface forces acting at the interface between the cantilever tip and the sample, without physical interaction. The key advantage of this approach is that it eliminates the forces exerted by the tip on the sample, thereby preventing potential mechanical damage or destruction to the sample [65,66,70,71].

*Tapping mode* operates through dynamic actuation, involving intermittent contact between the cantilever tip and the sample surface. This mode serves as a hybrid between the contact and non-contact methods, providing a semi-contact interaction. Tapping mode reduces lateral forces, which significantly minimizes the risk of damage to the sample compared to contact mode. In this mode, both height and phase information are obtained simultaneously by detecting changes in amplitude and frequency, and the phase difference between the generated and received signals [72].

The choice of AFM operating mode has a direct impact on the quality and performance of the images obtained. For instance, in the contact mode, biological samples can be highly fragile, and direct interaction with the cantilever tip may cause damage due to frictional forces. This could lead to the breakage or degradation of the biological material [66]. In contrast, tapping or non-contact modes are less likely to cause such damage and are therefore preferred for analyzing delicate biological samples, ensuring both high-quality imaging and the preservation of sample integrity [61]. These modes provide clearer, undisturbed images and are particularly advantageous for analyzing fragile or sensitive biological materials.

Although the AFM operating modes, contact, non-contact, and tapping (semi-contact), have significant importance, it is important to remember the relevance of each in the context of the analysis of biological specimens in liquid media. Under such conditions, non-contact and tapping modes can become less effective due to the mechanical instability generated by the vibrations of the lever. These vibrations can lead to the detachment of cells from the substrate, especially in the case of magnetotactic bacteria, which have a relatively weak adhesion to the analysis surface. As a result, samples can be removed from the scanning field by fluid movement or by forces induced by cantilever oscillations. In contrast, the contact mode, although more invasive, ensures a stable mechanical interaction between the microscope tip and the bacterial surface, which makes it more suitable for obtaining high-resolution images in liquid media, without compromising sample positioning. The choice of working mode thus becomes an essential factor for the success of AFM experiments involving working with living specimens, and compatibility with the liquid environment is a key advantage compared to electronic techniques [66]. To date, atomic force microscopy and magnetic force microscopy have been successfully applied to the study of various classes of living samples, including bacteria (such as *Escherichia coli* (*E. coli*) [73]), eukaryotic cells (fibroblasts, epithelial cells, stem cells) [74], yeasts [75], and even unicellular algae [76]), demonstrating the versatility of these techniques in investigating biological structures under conditions close to physiological ones.

AFM proves to be an indispensable tool for imaging, characterizing, and evaluating the properties of nanostructured and biological materials, including magnetotactic bacteria. This is particularly true when it comes to understanding the unique characteristics of MTB, which are essential for exploring their potential applications, such as in drug delivery systems, tumor degradation, and other biocompatible uses.

A notable example of AFM utility in the study of MTB is the work conducted by Lefèvre et al. (2009) [77] on *Magnetococcus* sp. MC-1, a species isolated from the Mediterranean Sea. Using AFM with the Topometrix TMX2000 Explorer (Topometrix Corporation, Santa Clara, CA, USA), the researchers performed imaging in tapping mode on MC-1 cells adsorbed on V-1 muscovite treated with uranyl acetate (0.1%). They also employed Nile red staining to visualize lipid storage granules (1–2 per cell). Despite its ubiquitous distribution in marine and freshwater environments, *Magnetococcus* sp. MC-1 is difficult to isolate and culture due to its preference for chemical gradients in stratified sediments, which are challenging to replicate in laboratory conditions. As a result, only a limited number of strains have been isolated in pure culture [77].

One well-established aspect of magnetotactic bacteria is their magnetotactic behavior, which manifests in two types of magnetotaxis: polar and axial. Axial magnetotaxis enables the bacteria to move in both directions along the magnetic field, while polar magnetotaxis has been observed to cause the bacteria to move persistently in one direction along the magnetic field [78].

Researchers analyzed MTB MC-1 cells using multiple microscopy techniques, which helped to characterize and reveal key features of the strain. Through transmission electron microscopy (TEM), they observed that the individual flagella of MC-1 are located exclusively at the distal ends of the cell. Furthermore, atomic force microscopy revealed that the flagella in each bundle are encased in a common sheath. Flagella serve as the primary means of locomotion for these bacteria, and Lefèvre et al. (2009) [77] discovered that the flagellar sheath was not present in other MTB strains. The identification of this sheath through AFM was complemented by additional techniques to further analyze and assess the structure, function, and role of the flagella in MC-1 locomotion [77]. For instance, in the area of identifying new strains (Table 5), Salam et al. (2023) isolated a new strain of MTB, *Magnetospirillum moscoviense* MS-24, isolated from Banjosa Lake, Pakistan, which was characterized using AFM, high-resolution scanning electron microscopy (HR-SEM), and TEM to observe magnetotaxis movement [29].

In another study, researchers utilized two AFM systems in parallel for the analysis of MTB. The equipment included the Digital Instruments Bioscope AFM with NanoScope IV controller (Digital Instruments, Santa Barbara, CA, USA) and the Asylum Research MFP3D AFM (Asylum Research, Santa Barbara, CA, USA), both integrated with inverted optical microscopes (Zeiss Axiovert 200M, Oberkochen, Germany, and Nikon 300TE, Tokyo, Japan). These setups allowed precise positioning of the cantilever tip over the sample, facilitating high-resolution imaging. The AFM tips used were of high quality, such as the Olympus (Tokyo, Japan) AC160TS and AC240T, with tip rays of approximately 10 nm, and silicon nitride probes from Bruker (Billerica, MA, USA) (DNP-10), with tip rays ranging from 20 to 60 nm [79].

The experiments were conducted using a saline solution (PBS, pH 7.4) under ambient conditions, at room temperature and 40% relative humidity. The magnetotactic bacteria strain *M. gryphiswaldense* MSR-1 was used for these studies. AFM was employed to acquire height images, providing detailed topographical information, specifically about the lateral and vertical dimensions of the bacterial cells. In parallel, amplitude images were also captured, which highlighted the edges and distinctive features of the MTB. The AFM images revealed intracellular inclusion bodies with diameters of approximately 250 nm, as well as the magnetosome chain, which consisted of about eight magnetosomes, each around 75 nm in size, and their alignment within the bacteria. Furthermore, the flagellum was identified and analyzed, with its thickness estimated to be approximately 100 nm [79].

Another research group analyzed samples of the *Magnetospirillum moscoviense* MS-24 strain, which was collected from aquatic sediments in Lake Banjosa, Rawalakot, Pakistan, using AFM. This study revealed the spiral shape of the bacteria and provided a three-dimensional structural analysis, highlighting the bacterial magnetosomes [29].

All these studies illustrate how AFM can reveal previously unseen details about MTB morphology, behavior, and internal structures, contributing to a deeper understanding of their magnetotactic properties and supporting their potential applications in biotechnology and medicine.

From a different perspective, one of the earliest characterizations of the magnetic moment of MTB using AFM was conducted by Zahn et al. (2017) [80] during their study of the *Magnetospirillum gryphiswaldense* strain. Their analysis utilized magnetic tweezers [80]. In contrast, Marcano et al. (2022) examined the magnetic anisotropy of individual magnetosomes in the *Magnetovibrio blakemorei* MV-1 strain [47].

### 5.4. Magnetic Force Microscopy in the Analysis of MTB

A more recent study (2024) [48] stands out for its use of magnetic force microscopy (MFM) within atomic force microscopy. MFM is a technique specifically designed for magnetic characterization at the nanoscale with a high spatial resolution of 20 nm [48,81]. This method is primarily employed to characterize nanostructures and thin films, owing to its high sensitivity to surface signals. However, the parasitic fields produced by the MFM probes, along with the distance between the probe tip and the sample, are critical factors that can impact the accuracy of the results. One notable advantage of MFM is its flexibility, allowing customization of the magnetic probes, modification of the sprayed (Fe, Co, Ni, Au, C, Ag) coating, creation of a magnetic vortex core, or the addition of a ferromagnetic element to the tip, making it adaptable to specific investigative needs, as shown in Table 6.

Marqués-Marchán et al. (2024) [48] used AFM to analyze the *M. gryphiswaldense* MSR-1 strain, which was cultured on standard DMSZ medium (DMS 6631) and fixed with glutaraldehyde. Their study revealed that the magnetosomes were aligned along the crystallographic axis in a chain formation. Cryotomographic imaging further demonstrated that the magnetosome chain has a helical structure, with the number of magnetosomes varying between 10 and 30. Unlike other characterization methods, the researchers found that MFM enabled the observation of the individual characteristics of single magnetosomes. Additionally, the application of an external magnetic field, in combination with MFM, allowed for a more detailed analysis of the magnetization process within the chain, providing further insights into the coercive fields of magnetosomes [48].

Winkler et al. (2023) [69] also reviewed *M. gryphiswaldense* strain characterization, focusing on the use of MFM probes to assess the magnetic response of the bacteria. They found that most measurements of the magnetic properties of the strain were in a detection limit in the range of 3.8–4.5 nm [69].

An essential aspect in the evaluation of microscopic techniques used in biological and biomedical research, especially in the case of magnetotactic bacteria, is the ability to work with live specimens. This feature becomes even more relevant in the context of comparison with electron microscopy, which, although offering extremely high resolution, imposes vacuum conditions and invasive preparation, incompatible with the study of living organisms. In contrast, atomic force microscopy and, complementarily, magnetic force microscopy offer the possibility of examining magnetotactic bacteria in the living state, under conditions close to their natural environment. This capability transforms them into valuable tools for non-destructive structural and functional analysis, allowing the real-time observation of topographical or magnetic changes without compromising cellular viability [70]. Both AFM and MFM can be used to study magnetotactic bacteria in the living state, with certain well-controlled experimental conditions. Unlike electron microscopy techniques, which require vacuum and fixed or dehydrated samples, AFM and MFM can operate in liquid media, which allows them to investigate living bacteria under conditions close to physiological ones. To provide a comprehensive overview of the key aspects of using AFM or MFM, we refer to the main advantages and disadvantages of each technique, which are summarized in Table 7. In addition, Table 8 summarizes the MTB strains studied by AFM and the main characteristics identified.

### 5.5. Spectroscopic Methods for MTB Investigations

The optical characterization of magnetotactic bacteria is a fundamental approach in the direction of capitalizing these microoragnisms as functional biological resources. The information obtained from these analyses contributes to a thorough understanding of their specific properties, such as their internal organization, biochemical composition and magnetic behavior, essential aspects for their controlled integration into technological systems. In this context, detailed characterization is not only an end in itself but also a starting point in designing smart detection platforms, in which MTB or their magnetosomes are used as active elements. Knowing these features allows the correlation of the structure with the functionality, facilitating the development of new generation biosensors, adapted to various analysis requirements. Thus, these data become essential in the elaboration and optimization of some acoustic, optical, electrochemical, or magnetic biosensors, which can respond with high sensitivity and selectivity to relevant biological or chemical stimuli, early diagnosis, or in monitoring environmental parameters. This characterization stage is, therefore, the critical link between the fundamental exploration of the MTB and their implementation in innovative detection systems.

**Figure 9:**
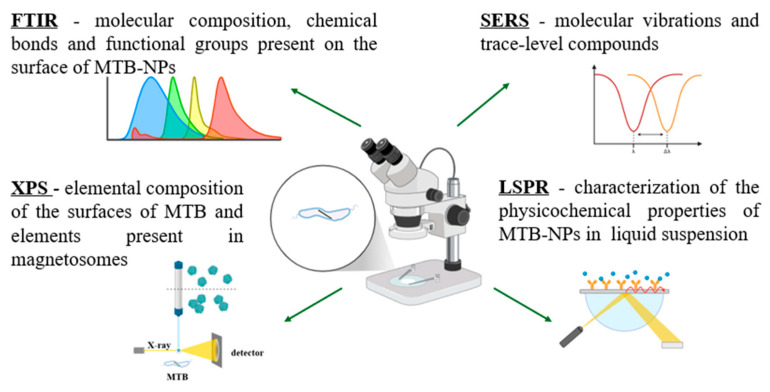
Main characteristics of MTB-NPs evaluated using spectroscopy techniques.

The main characteristics of MTB-NPs are often analyzed with spectroscopy techniques (Figure 9).

#### 5.5.1. Infrared Spectroscopy, Fourier-Transform Infrared

Fourier Transform Infra-Red Spectroscopy (FTIR) is a spectroscopic technique that measures the absorption of infrared light and provides information about the molecular composition, chemical bonds, and functional groups present on the surface of magnetic nanoparticles. FTIR is useful in studying the surface functionalization of nanoparticles as different functional groups absorb different wavelengths of infrared light, which translates into characteristic peaks in the spectrum that are then converted into raw data via the Fourier transform [82].

Infrared spectroscopy, especially Fourier-Transform Infrared, has been used to identify organic functional groups in the bacterial envelope and on magnetosome membranes, such as amide, carboxyl, and phosphate groups. This technique provides important information about the biochemical environment surrounding magnetosomes. In the future, IR spectroscopy could be used to monitor metabolic changes during biomineralization or to analyze surface modifications of magnetosomes intended for biomedical use [82].

#### 5.5.2. X-Ray Photoelectron Spectroscopy

Another useful method in the study of magnetic particles is X-ray photoelectron spectroscopy (XPS). This method plays a role in characterizing the elemental composition of the surfaces of microbial cells and implicitly in identifying the elements present in magnetosomes, such as iron, oxygen or sulfur [83].

Shimoshige et al. (2017) [83] performed XPS analyses on the magnetotactic bacterial strain *Magnetospirillum magneticum* strain RSS-1 to determine the oxidation states of metal ions. This was possible by checking the presence of iron, oxygen, and sulfur in and on magnetic nanoparticles extracted from the RSS-1 strain [83]. XPS analysis makes it possible, in addition to elemental analysis, also to check for possible contamination of magnetosomes with other organic or inorganic substances [84].

X-ray photoelectron spectroscopy has proven crucial for analyzing the elemental composition and oxidation states of iron in magnetosomes, distinguishing between Fe^2+^ and Fe^3+^ and confirming the presence of magnetite (Fe_3_O_4_) or greigite (Fe_3_S_4_). XPS has also been used to detect surface-bound elements like oxygen, sulfur, or phosphorus. Looking ahead, XPS could be applied in the optimization of magnetosome extraction and purification processes, as well as in characterizing surface functionalization strategies for targeted drug delivery or imaging applications [83,84].

#### 5.5.3. Localized Surface Plasmon Resonance

The localized surface plasmon resonance (LSPR) method has been used to develop simple and rapid methods for the identification of *Vibrio cholerae*, considered the cause of numerous epidemics and pandemics of intestinal diseases [85].

LSPR is an optical phenomenon used in nanotechnology that occurs as a result of the interaction of light with metallic nanoparticles (such as gold or silver). The interaction determines the collective oscillation of free electrons on the surface of the nanoparticles at a certain frequency.

This method has been successfully used in the design of a LSPR-based colorimetric aptasensor using gold nanoparticles for the identification of *V. cholerae* [86]. LSPR has been used to study the effect of magnetic nanoparticles. A study conducted in this regard demonstrated that magnetic nanoparticles (Fe_3_O_4_) significantly enhanced the localized surface plasmon resonance of metal nanoparticles [87]. MNP acts as a signal amplifier for the plasmonic response due to the high refractive index, high molecular weight, and superparamagnetic properties of Fe_3_O_4_ particles [87]. Moreover, surface plasmon resonance sensors can serve as effective tools in characterizing the physicochemical properties of magnetic particles in different liquid suspensions (distillated water, phosphate-buffered saline solution), as demonstrated in the study by Mostufa et al. (2025) [88].

Localized surface plasmon resonance has been primarily applied to investigate the interaction of magnetosome surfaces with surrounding molecules by tracking changes in the plasmonic response of nearby metallic nanostructures. Although less common than other techniques in MTB studies, LSPR holds great potential for label-free detection of biological interactions involving magnetosomes, such as protein binding or cell targeting. It may be further developed for real-time, in situ biosensing applications involving MTB-derived nanomaterials [87,88].

#### 5.5.4. Surface-Enhanced Raman Scattering

Another advanced spectroscopic technique that has found its application in magnetic particle analysis is surface-enhanced Raman scattering (SERS). This technique amplifies the Raman signal of molecules placed on nanostructured metal surfaces (such as gold, copper or silver), allowing researchers to study molecular vibrations and identify trace-level compounds (10^5^–10^0^ cells/mL) with very high sensitivity down to the level of a single molecule [89,90].

Cheng et al. (2024) [91] attempted to develop a biosensor for the rapid and broad-spectrum detection of bacteria for effective clinical monitoring. The biosensor was developed for the rapid and sensitive detection of *S. aureus* and *P. Aeruginosa* [91]. The biosensor uses magnetic nanoparticles modified with specific antibodies as capture probes. Magnetic separation of antibodies, respectively, the application of SERS has proven a significant improvement in the detection capacity of the biosensor [91]. SERS has been effectively used to detect biomolecules associated with magnetotactic bacteria with high sensitivity, including proteins, lipids, and DNA components found near or on magnetosome surfaces.

In the future, SERS could be employed in real-time monitoring of magnetosome biosynthesis and functionalization [92], or in developing highly sensitive MTB-based biosensors for medical diagnostics [93] or environmental detection [94].

Considering the aforementioned aspects, in Table 9, we have summarized the main advantages and disadvantages of spectroscopy techniques that can be used to analyze and characterize magnetotactic bacteria.

## 6. UltrasSensitive Biosensors Using Whole-Cell Magnetotactic Bacteria vs. Their Magnetosomes

**Figure 10:**
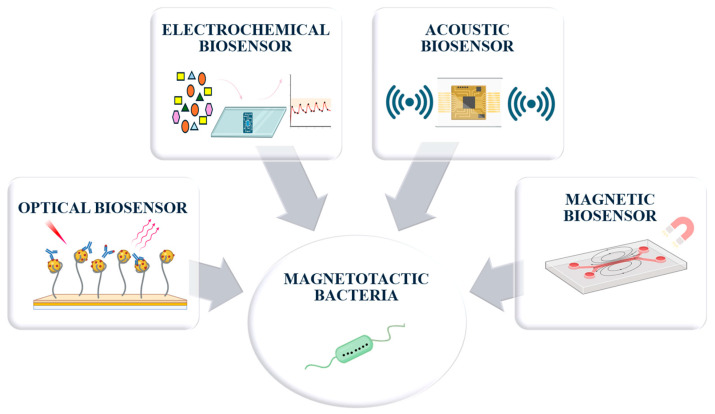
Types of biosensors in which magnetotactic bacteria can be used.

Comprehensive characterization of MTB is a fundamental requirement for the use of these microorganisms as functional biological resources. The information obtained by these surface analysis techniques contributes to a deeper understanding of their specific properties, such as their internal organization, biochemical composition, and magnetic behavior, which are essential aspects for their controlled integration into technological systems. In this context, detailed characterization is not only a goal in itself but also a starting point for the design of smart sensing platforms in which MTB or their magnetosomes will serve as active elements. Knowledge of these properties allows researchers to link structure and functionality, thus facilitating the development of next-generation biosensors to different analytical requirements. Moreover, these data are essential for the development and optimization of different types of biosensors (e.g., acoustic, optical, electrochemical or magnetic) able to respond with high sensitivity and selectivity to complex biological or chemical stimuli, relevant for early diagnosis or monitoring of environmental parameters, by establishing a direct biocompatibility link with MTB (Figure 10).

### 6.1. Acoustic Sensing

Acoustic sensors have evolved as a feasible and versatile method for detecting, interpreting, and monitoring sound variations at the interaction with different physical environments. Using the characteristics of propagation of acoustic signals, these biosensing instruments have found utility in various applications (such as biomedical or environmental field) for monitoring, diagnosis, or identification of parameters [95,96,97,98]. The acoustic detection, in the case of these tools, is based on the analysis of the sound variations that, depending on the specificity of the application, can facilitate the identification of some changes in the properties of the materials or can detect the presence of substances or materials [95].

For instance, in the biomedical field, acoustic sensors are used for the qualitative and quantitative analysis of antibiotics. More precisely, they are used to study the phenomena that occur at the solid–liquid interface by associating the analytes under analysis with changes in the parameters of the propagated acoustic waves [99,100].

Over time, they have become the central piece in the construction of miniaturized and portable devices for performing various analyses (food, environmental, or even health care) [101]. They use acoustic waves generated by various piezoelectric materials (e.g., zinc oxide, silicon oxide, or lead-zirconate-titanate) in order to obtain information about different analytes (e.g., environmental gases, vapors, proteins, antibodies, glucose, pesticides, heavy metals) [102]. Depending on the type of propagation of mechanical waves in piezoelectric materials, acoustic sensing devices follow two main directions, namely surface acoustic waves (SAW) and bulk acoustic waves (BAW) [97,100,103,104].

A recent example of the use of acoustic sensors is the case of the determination of ampicillin in conductive solutions [98]. Following the intensive use of antibiotics in the field of medicine, Borodina et al. (2022) [98] conducted an experimental study using a microbial cell-based sensor to determine ampicillin in conductive liquids. The aim of their study was addressing the growing concerns about the contamination of environmental resources, especially water, as a result of the global problems that the consumption of these drugs can cause. In this regard, they used a system of acoustic sensors (based on lithium niobate) and microbial cells of *E. coli*, strain K-12, as the main element for the detection of the antibiotic (ampicillin) in different concentrations (2, 5, 8, 12, 15, and 18 µg/mL). The results obtained by them indicated the possibility and usefulness of detecting antibiotics from conductive liquids (buffer solution), under conditions of high conductivity of the measurement medium, emphasizing the ability of this approach to analyze specific substances directly in the liquid [98].

### 6.2. Electrochemical Sensing

Electrochemical sensors are a cornerstone of modern analytical methods due to their versatility and sensitivity in detecting a wide range of biological, chemical, or toxicological analytes. Their sensitivity, rapidity of analysis, miniaturization possibilities and low production costs have propelled them among the indispensable devices in various sectors of activity. For example, we mention applications for monitoring and evaluating food safety, industrial product control, environmental and water monitoring applications, as well as their utility in the field of clinical and toxicological diagnostics [105,106,107,108].

This type of sensor detects and converts chemical information (physical properties of analytes or information generated following a chemical reaction) into an analytical signal [109,110], and the main elements of such a device are the receptor and the physicochemical transducer [110].

The receptors used in the construction of electrochemical sensors vary depending on the materials used. In this regard, different materials, nanomaterials (copper, nickel, oxides of non-transition metals) [111], conductive polymers (polyaniline, polypyrrole, polythiophene) [111], or carbon-based composites can be used (carbon nanotubes, carbon paste electrodes, single-walled carbon nanotubes, or multi-walled carbon nanotubes) [112].

In environmental applications, electrochemical sensors have been successfully used to detect pollutants and environmental contaminants [113]. A recent review in this direction evaluated recent advances and highlighted the usefulness of metal oxides in this regard, highlighting the sensitivity and selectivity of their use [114].

In ensuring food safety, electrochemical sensors also play a critical role by detecting different types of contaminants (like toxins, mycotoxins or pesticides) [114], and in the medical field, they have facilitated the development of the non-invasive diagnostic branch by allowing the analysis of various substances (e.g., glucose, uric acid, alcohol, metabolites) present in biological fluids (blood, serum, saliva, plasma, interstitial fluid, tears, sweat) [115,116,117,118]. The development of sensors that can detect analytes with high sensitivity and stability has allowed their use in the analysis of numerous biomarkers (troponin, myoglobin, procalcitonin, C-reactive protein) [119,120].

In this context, we consider that magnetotactic bacteria would be promising candidates for the construction of electrochemical sensors (Figure 10). As a result of the fact that they are environmentally friendly, both the magnetosomes and the nanoparticles contained by MTB (magnetite/greigite) could prove increased effectiveness in capturing heavy metals or different contaminants from waste water, soil, food, and even from biological fluids. Evidence to support this statement are studies that have analyzed the properties and potential applications of these natural nanoparticles mineralized by MTB [13,121,122,123,124,125,126,127].

### 6.3. Optical Sensing

As with acoustic and electrochemical sensors, optical sensors were built to identify changes or variations, but in this case, they detect changes in the properties of light (polarization, wavelength, or intensity) resulting from interactions with target analytes [128]. These changes are then converted into electrical signals for analysis. The main mechanisms include absorption [129,130], reflection and scattering [131,132,133], fluorescence and phosphorescence [134], and surface plasmon resonance [135,136,137]. Their ability to provide highly sensitive information, as well as non-invasive analysis and real-time data acquisition, has made their use integrated in various sectors of activity (industrial sector, environmental monitoring, and even in medical diagnostic applications) [132,138,139].

A very recent review on optical sensors explored the latest advances in optical sensing techniques to assess their role in noninvasive transdermal biomarker detection [140]. The transformative potential of optical sensors is a useful signal in transforming, adapting, and improving them so that they can be used in the diagnosis of various conditions and even in promoting proactive management of the prevention of various diseases. The authors of this study highlighted the importance of overcoming current challenges related to user comfort during long periods of sensor wear. This can be achieved, according to the data they collected, by shifting the focus to the development and use of advanced materials in close collaboration with nanotechnology techniques to improve the sensitivity and specificity of these sensors [140].

Until now, optical sensors have been studied to demonstrate their ability to detect and measure transcutaneous biomarkers from sweat (hormones—cortisol [141], ethylic alcohol [142], pH changes [143,144]) or gases that diffuse through the skin (oxygen, carbon dioxide, and other gases that are indicative of metabolic activity or dysfunction) [145,146,147].

In addition to medical applications, this type of sensor has also found utility in environmental monitoring applications. As an example, we refer to the sensitivity and specificity of these sensors, in wearable devices, in the recognition of bio-contaminants [148] or pathogens demonstrated in several studies in recent years (*E. coli* and *S. aureus* [149], SARS-CoV-2 [150]). In this sense, we recognize a potential utility of magnetotactic bacteria in the construction of optical biosensors (Figure 10) for the purpose of identifying substances in different biological fluids or environmental monitoring applications [151,152,153]. Such studies support the future direction of study and applicability of magnetosomes and MTB.

### 6.4. Magnetic Sensing

Biosensors are self-contained integrated devices that convert a biological response into a measurable signal [154]. They represent a rapidly growing class of analytical devices that allow fast detection and quantification of specific substances [155]. Since their discovery in 1962 [156], biosensors have experienced an upward slope in terms of evolution, development and especially utility, becoming indispensable tools in various fields and sectors of activity, such as environmental monitoring, food safety, biotechnology, and medical diagnosis [157,158]. The main component is one of a biological nature (antigens, DNA, RNA, enzymes, antibodies, microorganisms) complemented by an electronic sensing element (electrochemical, optical, thermal, or piezoelectric) [155,159,160]. The specificity of the biological component in combination with physical measurements allow biosensors to obtain responses characterized by a high degree of sensitivity, selectivity and rapidity [161]. Over time, numerous such devices have been created, developed, and tested, including their impact and effectiveness on human health [158,159].

Bacterial infections and inappropriate administration of antibiotics have rapidly led to the development of pathogens resistant to antimicrobial therapies, and in this direction, biosensors, micro- and nanorobots have an exponential potential in the prevention, treatment, and diagnosis of infections. They open the opportunity to manage infections at different stages and can thus contribute to the effective monitoring and treatment of infections. However, one of the main challenges lies in the navigation of micro/nanorobots in biological environments [162].

The unique characteristics of magnetotactic bacteria give them an amazingly promising potential for incorporation and use in biosensors, as well as in micro- or nanorobots. An example in this regard was the use of MTB in biosensors for the detection of food contaminants. The increasing number of food poisoning infections and digestive disorders caused by *E. coli* has gradually become a worrying problem. In this regard, a group of researchers demonstrated the applicability of a biosensor for the detection of *E. coli* in contaminated food samples. The biosensor consisted of a complex of magnetosomes coupled with specific antibodies for the detection of antigenic polysaccharides (LPS) of *E. coli* [163].

The electrochemical studies they used demonstrated the effectiveness of the biosensor in detecting LPS in a wide range of concentrations (0.5–50 μg/mL) in food products (water, milk, pineapple juice) [163]. For examples, in order to analyze the commercial applicability of the developed sensor, the authors performed electrochemical studies using screen-printed carbon electrode (SPCE), commonly used electrode for electrochemical biosensor application. The latter are feasible for single use, cheap, and efficient for small volume quantification of samples (food, dietary supplements, or different body fluids) [164]. The magnetosome-based biosensor detected low concentrations (0.5 μg/mL) of LPS considering the small volume (10 μL) of the complex (magnetosome-antibody), respectively, in the low LPS concentration in the analyzed samples (1.6 μg/mL) applied on the SPCE. Thus, they concluded that the SPCE is a cost-effective, stable, and useful system due to its portability and the possibility of analysis in medical analyses, but also in environmental analyses, being easily transported to the site [163].

Bacterial nano- and microrobots are a rapidly emerging context in the field of medical diagnostics, but their realization in such a way that they meet the requirements of controlled motility and specific functions remains a significant challenge [165,166]. Currently, diagnostic bacterial microrobots are prepared by standardized synthetic biology methods and by carefully controlled genetic engineering and manipulation techniques (e.g., CRISPR-Cas9) [167]. Their use in conjunction with synthetic nanoparticles is an aspect that still requires further studies due to their in vivo behavior, toxicity, reactivity, and their effects on the body in the medium and long term [168,169]. An important property of magnetosomes, in this regard, is the possibility of modifying their properties (through chemical modifications or genetic manipulation), which may make it possible to implement analytical protocols that allow their use in such devices [169].

Chen et al. (2014) [170] developed a microrobot based on magnetotactic bacteria (Magnetotactic ovoid strain MO-1) coupled with rabbit polyclonal antibodies (anti-MO-1), which they applied for the isolation of Staphylococcus aureus. Their study demonstrated the possibility of using bacterial microrobots carrying pathogens in a microfluidic chip, they were capable of transporting S. aureus to a target point (responsible for the magnetic zone). Thus, the potential of MTB in the use of self-propelled, magnetically guided microrobots was highlighted for the purpose of isolating pathogens [170].

In subsequent studies [171,172], researchers explored the possibility of neutralizing *S. aureus*, both in vitro and in vivo, by magnetic hyperthermia mediated by MTB (MO-1 coupled with polyclonal anti-MO-1 antibodies). Suspensions formed by the MTB-antibody complex to which a suspension of *S. aureus* was added were subjected to an alternating magnetic field. Their study demonstrated an efficiency of hyperthermia of up to 50% in neutralizing pathogens, and in vivo experiments (on murine models) demonstrated that magnetic hyperthermia mediated by antibody-coated MTB significantly improved wound healing, suggesting the usefulness of microrobots in the treatment of skin infections, respectively wounds induced by *S. aureus* [171,172].

In a more recent study, Song et al. (2023) [173] combined the autonomous self-propulsion of *Magnetospirillum magneticum* strain AMB-1 with magnetically controlled maneuvering to create a biohybrid microrobot for the removal of pollutants from water. This aspect allowed for precise directional control of the bacteria (placed in river water solutions) by modulating the value of a clockwise rotating magnetic field and made it possible to highlight properties such as their motility in aqueous solutions, and rapid response to directional control by a magnetic field, and especially their ability to incorporate pollutants (chlorpyrifos pesticide) [173].

## 7. Discussions

Characterization of bacteria capable of aligning and moving along magnetic field lines relies on the advanced microscopy techniques [45,47,48]. The membrane bound crystals of magnetite and greigite, synthetized by magnetetotactic bacteria, are usually arranged in chains and function as a biological compass [82,174,175]. In order to study both the morphological features of the cells and the ultrastructure and properties of magnetosomes and magnetic nanocrystals, a combination of different microscopy techniques is required. Initial observations of MTB often begin with conventional light microscopy [13,176,177], which provides a general overview of cellular morphology and enables the visualization of their motility in response to magnetic fields. While basic light microscopy lacks the resolution to reveal subcellular details, techniques such as phase-contrast [178,179] and differential interference contrast (DIC) microscopy [180,181,182] significantly improve image quality by enhancing contrast without staining, making them ideal for examining live cells in more detail. These approaches allow researchers to track the unique magnetotactic behavior and identify morphological variations among different strains.

For investigations of the internal structures of these bacteria and specifically the organization and morphology of magnetosomes, transmission electron microscopy is a critical tool. TEM offers nanometer-scale resolution, allowing the visualization of individual magnetosomes, their crystal shapes, arrangements within the cell, and their membrane-bound nature. When coupled with techniques like energy-dispersive X-ray spectroscopy [183], TEM also provides insights into the elemental composition of the magnetosomes, while high-resolution transmission electron microscopy (HRTEM) [26] enables detailed examination of the crystals. Complementing TEM, scanning electron microscopy is used to study the external morphology of the cells. SEM provides detailed three-dimensional images of the cell surface. Moreover, atomic force microscopy offers another layer of analysis by enabling topographical mapping of the bacterial surface at nanometer resolution [70]. AFM is particularly valuable because it can operate under near-physiological conditions, preserving the natural state of the cells. It also allows the measurement of mechanical properties of the bacterial surface, and in magnetic force mode, it can help reveal the magnetic characteristics associated with magnetosome chains.

Additionally, in cases where molecular or protein-level information is needed, fluorescence microscopy [184], especially when combined with genetic engineering or specific staining protocols, becomes a powerful tool. Confocal laser scanning microscopy [58], in particular, allows the acquisition of high-resolution, three-dimensional images of fluorescently labeled bacterial cells and can be used to localize proteins involved in magnetosome formation and cellular organization [58].

SERS, LSPR, IR, and XPS offers a powerful toolkit of spectroscopic techniques for the in-depth analysis of magnetotactic bacteria. SERS and LSPR are particularly effective for ultrasensitive molecular detection and probing surface interactions [185,186,187], while IR spectroscopy helps identify organic functional groups [188,189,190], and XPS provides detailed insights into elemental composition and oxidation states [191,192]. Combined, these tools greatly enhance our understanding of biomineralization processes, facilitate precise characterization of bacterial nanostructures, and support the development of magnetosome-based applications in fields such as biomedicine, environmental monitoring, nanotechnology, and theranostics [92,193,194].

One of the applications of great interest of biosensors is definitely in medicine. The use of the constituent cellular motility of MTB under the action of an external magnetic field for their controlled targeting, respectively the use of their remarkable properties is the basic idea in the development of micro/nanorobots and biosensors [165,167,168,169]. The development of these types of devices for drug transport or for the identification of targets for targeted treatments is a future direction with extremely promising potential. However, until this goal is achieved, there are many questions that arise regarding their route in the human body: How do MTB interact with different structures and components in the human body? How are they recognized by the cells of the immune system and how fast is this recognition? What are the medium and long-term effects of injecting MTB in different forms and combinations? How do they interact with other bacteria in the human microbiome? Is there a possibility that MTB can disrupt the bacterial microflora? Given that magnetosomes are known for their ability to capture minerals from their microenvironment, is there a possibility that they could unbalance minerals in the human body? These are just some of the questions that arise in this context, but in the future research we intend to conduct studies that, to the extent possible, help us elucidate the answer to some of them.

## 8. Conclusions

The study of magnetotactic bacteria greatly benefits from the application of multiple microscopy techniques, each contributing uniquely and with complementary insights into their structure, function, and magnetic properties. These techniques have not only deepened our understanding of MTB but have also paved the way for innovative applications in environmental monitoring, biotechnology, and nanotechnology; they collectively allow for a thorough investigation of MTB, from observing their magnetotactic behavior and overall cell morphology to probing the ultrastructure and composition of their magnetosomes at the nanoscale. Light microscopy and its advanced forms, such as phase-contrast and DIC, are invaluable for real-time, non-invasive monitoring of living cells, especially for studying motility and magnetic responsiveness. However, they are limited by their relatively low resolution, which prevents detailed visualization of subcellular structures.

TEM remains the gold standard for visualizing magnetosomes within the bacterial cytoplasm, offering high-resolution images that reveal crystal shape, size, and organization. Despite its exceptional detail, TEM requires complex sample preparation and operates under vacuum conditions, which can distort biological structures and consequently live imaging. SEM complements TEM by providing surface morphological information, but its inability to reveal internal structures without specific treatments is a notable limitation. SEM and TEM have elucidated the morphology and ultrastructure of MTB, including the complex cell wall architecture and the presence of magnetite crystals.

AFM introduces the advantage of imaging cells in near-native conditions, while also measuring surface topography and mechanical properties. AFM is pivotal in mapping the surface topography of MTB, revealing nanoscale features that influence bacterial adhesion and interaction with host cells. Nevertheless, its application is typically limited to surface features and relatively small scan areas, and it requires stable sample immobilization.

While AFM, TEM, and SEM provide essential structural and morphological data on magnetotactic bacteria and their magnetosomes, spectroscopic techniques such as SERS, LSPR, IR, and XPS offer crucial complementary insights into their chemical and molecular characteristics. SERS enables ultra-sensitive detection of biomolecules or magnetosome surfaces, while LSPR allows label-free monitoring of molecular interactions through plasmonic responses. LSPR-based sensors have been developed for the sensitive detection of MTB antigens, leveraging the unique optical properties of metal nanoparticles to enhance signal detection. IR spectroscopy identifies functional groups and tracks biochemical changes related to magnetosome membranes, and it has been employed to study the functional groups present in MTB complex lipid layers, aiding in the identification of biomarkers for diagnostic purposes. XPS offers in-depth analysis of the surface composition and iron oxidation states, providing valuable insights into the chemical characteristics of MTB surfaces. This technique has been crucial in identifying the elemental compositions and chemical states that are vital for understanding the mechanisms behind their resistance.

Finally, fluorescence and confocal microscopy offer powerful means to localize proteins and structures involved in magnetosome formation, especially when combined with genetic engineering or specific staining. These techniques depend heavily on the availability of suitable fluorescent markers and often suffer from lower resolution compared to electron microscopy.

Together, these techniques not only deepen our understanding of magnetosome biomineralization but also support the development of MTB-based applications in nanomedicine, biosensing, and environmental monitoring.

In summary, while no single microscopy method can fully capture the complexity of magnetotactic bacteria, the strategic combination of complementary techniques allows researchers to overcome individual limitations and build a comprehensive picture of MTB biology. This integrative approach is essential not only for advancing our understanding of magnetotaxis and biomineralization but also for exploring the biotechnological potential of these remarkable microorganisms.

This review was motivated by the need to provide a comprehensive overview of the methods used to characterize magnetotactic bacteria, with a particular focus on their analysis for potential applications in biosensor development. Through this review, we have managed to summarize and highlight the role, utility, and limitations of the different microscopy/spectroscopy techniques used in MTB characterization and analysis. Furthermore, we emphasize the importance of using these techniques in combination to have a more comprehensive and complete description of magnetotactic bacteria, their magnetosome mode, magnetic nanocrystal formation and exploration of their potential applications in biosensors, micro and nanorobots, biomedicine and theranostics.

## Figures and Tables

**Table 1:** Advantages and disadvantages of using TEM in MTB characterization.

Advantages	Disadvantages	Ref.
very high resolution (range)	complex sample preparation	[32]
ability to observe internal structures	high costs	[47]
localized chemical analysis	requires vacuum environment	[32]
atomic and nanometric scale imaging	risk of artifacts	[31]

**Table 2:** MTB strains characterized using TEM.

MTB Strain	TEM—Device Type	Characteristics Highlighted	Ref.
Magnetofossils	JEOL F200, 200 kV	morphology, magnetic components (detrital magnetic component)	[39]
Magnetofossils	JEM-2100Plus	composition of the magnetosome (magnetite)	[40]
*Magnetospirillum gryphiswaldense* MSR-1	JEM-2100 F, 200 kV	composition of the magnetosome (magnetite crystals), chain integrity, aggregation state of magnetosomes	[41]
*Magnetospirillum magneticum* AMB-1	JEOL JEM-1400 Plus, 120 kV	structural, morphological, and magnetic characteristics of the magnetosome chains (magnetite crystals)	[42]
*Magnetospirillum gryphiswaldense* MSR-1	JEOL JEM-14000 Plus, 120 kV	size (35–120 nm), shape (cuboctahedral), and arrangement of the magnetosomes (magnetite crystals)	[25]
*Magnetospirillum gryphiswaldense* MSR-1		size (40–80 nm), shape (cuboctahedral), arrangement (nonuniform distribution), and composition of the magnetosomes (magnetite crystals)	[43]
*Magnetospirillum gryphiswaldense* MSR-1	Jeol 2100F equipped with a Schotky FEG gun	characterization and organization of magnetosomes (magnetite crystals)	[44]
Magnetofossils	JEOL JEM-2100, 200 kV	morphological characteristics of the type magnetofossils (single-magnetic domain magnetite)	[45]
*Magnetospirillum gryphiswaldense* MSR-1	JEOL JEM-1400 Plus, 120 kV	internal arrangement, distribution, and chain analysis of type magnetosomes	[48].

**Table 3:** Magnetotactic bacterial strains characterized using SEM.

MTB Strain	SEM—Device Type	Characteristics Highlighted	Ref.
*Magnetospirillum moscoviense* MS-24	HR-SEM—FEI Quanta 250 FEG	magnetotaxis movement, size, and morphology of bacterial cells and magnetosomes	[29]
*M. blakemorei* MV-1	hybrid method—experimental and theoretical approaches STXM—MAXYMUS end station at HZB BESSY II, Berlin	magnetic anisotropy of magnetosomes	[47]
unidentified species of MTB	Zeiss (Jena, Germany) SUPRA 55VP field emission scanning electron microscope (FE-SEM)	information related to the morphology, physiology, or taxonomy of each cell	[58]

**Table 4:** Advantages and disadvantages of using SEM in bacteria characterization.

Advantages	Disadvantages	Ref.
high resolution and magnification, allowing the observation of very fine surface details	high cost	[59]
large depth of field	complex sample preparation	[53]
elemental analysis can provide detailed elemental composition data of the sample when paired with EDX	surface-only imaging	[29]
versatility and wide variety of materials (biological tissues, metals, polymers)	risk of sample damage	[47]
non-destructive imaging	requires skilled operators	[47]

**Table 5:** Advantages and disadvantages of using AFM in MTB characterization.

Advantages	Disadvantages	Ref.
high-resolution nanometric scale images	imaging depth limitation—offers high surface resolution, but it can be more difficult to obtain clear images of the internal structures of bacteria	[48]
observation of the fine details of bacteria’s structure, including their shape and size	complex operation and additional costs of maintenance	[67]
topographic analysis	relatively slow technique, obtaining a complete data set can take a long time	[68]
information about morphology and roughness	quantitative analysis of certain parameters may be more complicated and less precise compared to other techniques	[69]
measurement of mechanical properties	possible mechanical interference given by the interaction between the AFM tip and the bacterium; this can influence its morphology, which can lead to artifacts	[68]
evaluation of mechanical properties such as stiffness and elasticity of bacteria	useful for studying small bacteria, and its applicability may be limited when studying larger or multicellular organisms	[29]
does not require dyes or special coloration for sample preparation, thus reducing the risks of artifacts induced by these substances	sample artifacts—deformation, scratching, indentation	[67]
can be used to study living bacteria in an environment close to natural conditions, allowing real-time observations of their behavior		[63]
can be used to study both the morphology and physicochemical properties of bacteria		[64]

**Table 6:** Advantages and disadvantages of using MFM in MTB characterization.

Advantages	Disadvantages	Ref.
useful for studying bacteria that have magnetic particles	limited use for non-magnetic bacteria	[69]
provides high spatial resolution and can probe structures at the nanometer scale	only provides information about the surface of bacteria	[81]
useful for detecting fine magnetic features of bacterial cells	can be difficult to interpret because magnetic interactions can be influenced by various factors, such as the orientation of magnetic domains or external fields	[48]
provide insights into distribution, alignment, and morphology of MTB		[69]
non-destructive to the samples, preserving their integrity during analysis		[80]

**Table 7:** Comparison between AFM and MFM characteristics in MTB evaluation.

Property	AFM	MFM	Ref.
imaging focus	surface topography, mechanical properties	magnetic interactions and properties	[48]
resolution	high, nanometer-scale	high, but dependent on magnetic features	[81]
sample requirements	no special preparation required	must have magnetic materials (e.g., magnetosomes)	[48]
internal structure imaging	no, surface-level only	no, unless magnetic features are present	[81]
speed	relatively slow	slow	[70]
non-destructive	yes	yes	[65]
suited for	general surface properties, morphology, mechanical characteristics	magnetotactic bacteria, mapping magnetic domains	[81]

**Table 8:** MTB strains characterized using AFM, MFM, or magnetic imaging.

MTB Strain	Sample Preparation	Device Type/Description	Analysis Mode	Characteristics Highlighted with AFM/MFM	Ref.
*Magnetococcus* sp. MC-1	MC-1 adsorbed on V-1 muscovite treated with uranyl acetate (0.1%) and Nile red staining	Topometrix TMX2000 Explorer (Topometrix Corporation, Santa Clara, CA, USA)	Tapping mode	- flagellar sheath - lipid storage granules	[77]
*Magnetosprillum gryphiswaldense* MSR-1	saline solution, PBS, pH 7.4 in ambient atmosphere and at room temperature, with a relative humidity of 40%	Digital Instruments Bioscope AFM with controler NanoScope IV (Digital Instruments, Santa Barbara, CA, USA)	Tapping mode	- intracellular inclusion bodies with diameters of approximately 250 nm magnetosomes chain, approximately eight in number with dimensions of approximately 75 nm - positioning of the chain of magnetosomes within the MTB	[79]
		Asylum Research MFP3D AFM (Asylum Research, Santa Barbara, CA, USA)			
*Magnetosprillum gryphiswaldense* MSR-1	fixed bacteria suspended in the 85% (*v*/*v*) glycerol solution (dynamic viscosity of 135 mPas at 22.5 °C) placed on a glass coverslip (No. 1, 18 mm diameter, Marienfeld-Superior, Lauda-Königshofen, Germany) mounted into an aluminum holder	Magnetic tweezers (MT) setup with inverted light microscope (Nikon Eclipse Ti-U, Nikon, Tokyo, Japan) with a 20× magnification objective for calibration measurements (CFI Plan Achromat 20× objective, NA 0.4, Nikon) and a 60× magnification objective (CFI Plan Apochromat λ 60× oil objective, NA 1.40, Nikon)	Magnetic Tweezers	- quantifying the individual magnetic moments	[80]
*Magnetovibrio blakemorei* MV-1	DSM 18854, grown anaerobically at 30 °C in liquid medium; unstained cells adsorbed onto 300 mesh carbon-coated copper grids	X-ray Magnetic Circular Dichroism (XMCD), at room temperature, using ALICE station (Scienta Omicron, Berlin, Germany)	Magnetic imaging	- magnetic information on nanomagnets - magnetic properties - of anisotropic magnetic nanostructures	[47]
*Magnetospirillum gryphiswaldense* MSR-1	DMSZ: DMS 6631, cultured in flask standard medium containing 100 μM of Fe (III) citrate, at 28 °C, fixed with 2% glutaraldehyde standard MFM tips magnetic AFM probes with a sputtered magnetic coating (cobalt)	AFM and MFM experiments performed using a Nanotec Electronica S.L. measurements conducted in air and under liquid magnetic coil system used to perform Variable Field MFM (VF-MFM) measurements, with in-plane magnetic field (Nanotec Electronica S.L., Madrid, Spain)	Amplitude modulation mode	- local characterization of MTB - electrostatic charge present on MTB surfaces, repulsive - and attractive interaction between tip and sample - imaging of magnetosome chains inside bacteria detailed analysis of the chain magnetization process	[48]
*Magnetospirillum moscoviense* MS-24	growth culturing medium, specific and modified iron-containing medium for MTB growth; microfluidic chip was positioned on a microscope slide by plasma gas so that no air was present between the glass slide and chip; MS-24 inserted in the microfluidic chip by micropipette	Bruker ICON Dimension Microscope, bacterial broth culture suspended in phosphate buffer saline (PBS), pH 7.4, and placed the suspension onto a glass slide and air-dried, then placed the slide under a microscope (Bruker Corporation, Santa Barbara, CA, USA)	AFM	- bacterial spiral shape - three-dimensional structure of the sample presence of magnetosomes in the bacterial cell	[29]

**Table 9:** Comparison between FTIR, XPS, LSPR, and SERS spectroscopy techniques in MTB evaluation.

Spectroscopic Technique	Advantages	Disadvantages	Ref.
FTIR	- identifies functional groups on the magnetosome surface - non-destructive and rapid - useful for organic/membrane - analysis	- low spatial resolution - overlapping spectra from cellular components - limited sensitivity for trace materials	[82]
XPS	- provides detailed surface chemical composition - determines elemental oxidation states	- requires vacuum environment - imited to surface analysis	[83]
LSPR	- high sensitivity to changes in local refractive index - real-time monitoring of surface interactions - label-free detection	- depends on nanoparticle size, shape, and - environment-limited chemical specificity	[88]
SERS	- ultra-sensitive molecular fingerprinting - enhanced signal from surface-bound species - suitable for detecting low-abundance biomolecules	- requires nanostructured metallic substrates - signal reproducibility can be challenging	[94]

## References

[1] Lin W., Pan Y., Bazylinski D.A. (2017). Diversity and Ecology of and Biomineralization by Magnetotactic Bacteria. Environ. Microbiol. Rep..

[2] Bazylinski D.A., Lefèvre C.T., Schüler D., Rosenberg E., DeLong E.F., Lory S., Stackebrandt E., Thompson F. (2013). Magnetotactic Bacteria. The Prokaryotes: Prokaryotic Physiology and Biochemistry.

[3] Uebe R., Schüler D. (2016). Magnetosome Biogenesis in Magnetotactic Bacteria. Nat. Rev. Microbiol..

[4] Lin W., Bazylinski D.A., Xiao T., Wu L.-F., Pan Y. (2014). Life with Compass: Diversity and Biogeography of Magnetotactic Bacteria. Environ. Microbiol..

[5] Ali I., Peng C., Khan Z.M., Naz I., Sultan M. (2018). An Overview of Heavy Metal Removal from Wastewater Using Magnetotactic Bacteria. J. Chem. Technol. Biotechnol..

[6] Fuduche M., Davidson S., Boileau C., Wu L.-F., Combet-Blanc Y. (2019). A Novel Highly Efficient Device for Growing Micro-Aerophilic Microorganisms. Front. Microbiol..

[7] Dong Y., Li J., Zhang W., Zhang W., Zhao Y., Xiao T., Wu L.-F., Pan H. (2016). The Detection of Magnetotactic Bacteria in Deep Sea Sediments from the East Pacific Manganese Nodule Province. Environ. Microbiol. Rep..

[8] Sorty A.M., Shaikh N.R. (2015). Novel Co-Enrichment Method for Isolation of Magnetotactic Bacteria. J. Basic. Microbiol..

[9] Klumpp S., Faivre D. (2016). Magnetotactic Bacteria. Eur. Phys. J. Spec. Top..

[10] Rismani Yazdi S., Nosrati R., Stevens C.A., Vogel D., Davies P.L., Escobedo C. (2018). Magnetotaxis Enables Magnetotactic Bacteria to Navigate in Flow. Small.

[11] Le Nagard L., Morillo-López V., Fradin C., Bazylinski D.A. (2018). Growing Magnetotactic Bacteria of the Genus Magnetospirillum: Strains MSR-1, AMB-1 and MS-1. J. Vis. Exp..

[12] Liu J., Zhang W., Li X., Li X., Chen X., Li J.-H., Teng Z., Xu C., Santini C.-L., Zhao L. (2017). Bacterial Community Structure and Novel Species of Magnetotactic Bacteria in Sediments from a Seamount in the Mariana Volcanic Arc. Sci. Rep..

[13] Goswami P., He K., Li J., Pan Y., Roberts A.P., Lin W. (2022). Magnetotactic Bacteria and Magnetofossils: Ecology, Evolution and Environmental Implications. NPJ Biofilms Microbiomes.

[14] Paul N.L., Carpa R., Ionescu R.E., Popa C.O. (2025). The Biomedical Limitations of Magnetic Nanoparticles and a Biocompatible Alternative in the Form of Magnetotactic Bacteria. J. Funct. Biomater..

[15] Wang J., Xing Y., Ngatio M., Bies P., Xu L.L., Xing L., Zarea A., Makela A.V., Contag C.H., Li J. (2025). Engineering Magnetotactic Bacteria as Medical Microrobots. Adv. Mater..

[16] Ren G., Zhou X., Long R., Xie M., Kankala R.K., Wang S., Zhang Y.S., Liu Y. (2023). Biomedical Applications of Magnetosomes: State of the Art and Perspectives. Bioact. Mater..

[17] Yan L., Zhang S., Chen P., Liu H., Yin H., Li H. (2012). Magnetotactic Bacteria, Magnetosomes and Their Application. Microbiol. Res..

[18] Acosta-Avalos D., Leão P., Abreu F., Bazylinski D.A., Schmidt T.M. (2019). Magnetotaxis☆. Encyclopedia of Microbiology.

[19] Lefèvre C.T., Bazylinski D.A. (2013). Ecology, Diversity, and Evolution of Magnetotactic Bacteria. Microbiol. Mol. Biol. Rev..

[20] Faivre D., Böttger L.H., Matzanke B.F., Schüler D. (2007). Intracellular Magnetite Biomineralization in Bacteria Proceeds by a Distinct Pathway Involving Membrane-Bound Ferritin and an Iron(II) Species. Angew. Chem. Int. Ed..

[21] Tada Y., Yang P.C. (2019). Iron Oxide Labeling and Tracking of Extracellular Vesicles. Magnetochemistry.

[22] Wan J., Ji R., Liu J., Ma K., Pan Y., Lin W. (2024). Biomineralization in Magnetotactic Bacteria: From Diversity to Molecular Discovery-Based Applications. Cell Rep..

[23] Muñoz D., Marcano L., Martín-Rodríguez R., Simonelli L., Serrano A., García-Prieto A., Fdez-Gubieda M.L., Muela A. (2020). Magnetosomes Could Be Protective Shields against Metal Stress in Magnetotactic Bacteria. Sci. Rep..

[24] Ji R., Wan J., Liu J., Zheng J., Xiao T., Pan Y., Lin W. (2024). Linking Morphology, Genome, and Metabolic Activity of Uncultured Magnetotactic Nitrospirota at the Single-Cell Level. Microbiome.

[25] Jefremovas E.M., Gandarias L., Marcano L., Gacía-Prieto A., Orue I., Muela A., Fdez-Gubieda M.L., Fernández Barquín L., Alonso J. (2022). Modifying the Magnetic Response of Magnetotactic Bacteria: Incorporation of Gd and Tb Ions into the Magnetosome Structure. Nanoscale Adv..

[26] Li J., Zhang H., Menguy N., Benzerara K., Wang F., Lin X., Chen Z., Pan Y. (2017). Single-Cell Resolution of Uncultured Magnetotactic Bacteria via Fluorescence-Coupled Electron Microscopy. Appl. Environ. Microbiol..

[27] Basit A., Wang J., Guo F., Niu W., Jiang W. (2020). Improved Methods for Mass Production of Magnetosomes and Applications: A Review. Microb. Cell Factories.

[28] Peddie C.J., Genoud C., Kreshuk A., Meechan K., Micheva K.D., Narayan K., Pape C., Parton R.G., Schieber N.L., Schwab Y. (2022). Volume Electron Microscopy. Nat. Rev. Methods Primers.

[29] Salam M.A., Korkmaz N., Cycil L.M., Hasan F. (2023). Isolation, Microscopic and Magnetotactic Characterization of Magnetospirillum Moscoviense MS-24 from Banjosa Lake, Pakistan. Biotechnol. Lett..

[30] Egerton R.F. (2014). Choice of Operating Voltage for a Transmission Electron Microscope. Ultramicroscopy.

[31] Malatesta M. (2021). Transmission Electron Microscopy as a Powerful Tool to Investigate the Interaction of Nanoparticles with Subcellular Structures. Int. J. Mol. Sci..

[32] Li G., Zhang H., Han Y. (2023). Applications of Transmission Electron Microscopy in Phase Engineering of Nanomaterials. Chem. Rev..

[33] Tang C.Y., Yang Z., Hilal N., Ismail A.F., Matsuura T., Oatley-Radcliffe D. (2017). Chapter 8—Transmission Electron Microscopy (TEM). Membrane Characterization.

[34] Harris J.R. (2015). Transmission Electron Microscopy in Molecular Structural Biology: A Historical Survey. Arch. Biochem. Biophys..

[35] Franken L.E., Grünewald K., Boekema E.J., Stuart M.C.A. (2020). A Technical Introduction to Transmission Electron Microscopy for Soft-Matter: Imaging, Possibilities, Choices, and Technical Developments. Small.

[36] Bazylinski D.A., Garratt-Reed A.J., Frankel R.B. (1994). Electron Microscopic Studies of Magnetosomes in Magnetotactic Bacteria. Microsc. Res. Tech..

[37] Orue I., Marcano L., Bender P., García-Prieto A., Valencia S., Mawass M.A., Gil-Cartón D., Venero D.A., Honecker D., García-Arribas A. (2018). Configuration of the Magnetosome Chain: A Natural Magnetic Nanoarchitecture. Nanoscale.

[38] Zhang Q., Roberts A.P., Ge S., Liu Y., Liu J., Liu S., Tang X., Wang H., Wang D., Li J. (2022). Interpretation of Anhysteretic Remanent Magnetization Carriers in Magnetofossil-Rich Marine Sediments. J. Geophys. Res. Solid Earth.

[39] Kadam N., Badesab F., Lascu I., Wagner C.L., Gaikwad V., Saha A., Sangode S., Venkateshwarlu M. (2024). Discovery of Late Quaternary Giant Magnetofossils in the Bay of Bengal. Commun. Earth Env..

[40] Zhang X., Wang Y. (2024). Formation and Preservation Mechanisms of Magnetofossils in the Surface Sediments of Muddy Areas in the Yellow and Bohai Seas, China. Mar. Geol..

[41] Masó-Martínez M., Fryer B., Aubert D., Peacock B., Lees R., Rance G.A., Fay M.W., Topham P.D., Fernández-Castané A. (2023). Evaluation of Cell Disruption Technologies on Magnetosome Chain Length and Aggregation Behaviour from Magnetospirillum Gryphiswaldense MSR-1. Front. Bioeng. Biotechnol..

[42] Gandia D., Marcano L., Gandarias L., Villanueva D., Orue I., Abrudan R.M., Valencia S., Rodrigo I., Ángel García J., Muela A. (2023). Tuning the Magnetic Response of Magnetospirillum Magneticum by Changing the Culture Medium: A Straightforward Approach to Improve Their Hyperthermia Efficiency. ACS Appl. Mater. Interfaces.

[43] Pérez-Huerta A., Cappelli C., Jabalera Y., Prozorov T., Jimenez-Lopez C., Bazylinski D.A. (2022). Biogeochemical Fingerprinting of Magnetotactic Bacterial Magnetite. Proc. Natl. Acad. Sci. USA.

[44] Carvallo C., Fondet A., Le Fèvre R., Taverna D., Guyodo Y., Chebbi I., Dupuis V., Lagroix F., Khelfallah M., Guigner J.-M. (2023). Magnetic and Structural Properties of Biogenic Magnetic Nanoparticles along Their Production Process for Use in Magnetic Hyperthermia. J. Magn. Magn. Mater..

[45] Chen S., Yu M., Zhang W., He K., Pan H., Cui K., Zhao Y., Zhang X.-H., Xiao T., Zhang W. (2022). Metagenomic and Microscopic Analysis of Magnetotactic Bacteria in Tangyin Hydrothermal Field of Okinawa Trough. Front. Microbiol..

[46] Li J., Liu P., Menguy N., Benzerara K., Bai J., Zhao X., Leroy E., Zhang C., Zhang H., Liu J. (2022). Identification of Sulfate-Reducing Magnetotactic Bacteria via a Group-Specific 16S rDNA Primer and Correlative Fluorescence and Electron Microscopy: Strategy for Culture-Independent Study. Environ. Microbiol..

[47] Marcano L., Orue I., Gandia D., Gandarias L., Weigand M., Abrudan R.M., García-Prieto A., García-Arribas A., Muela A., Fdez-Gubieda M.L. (2022). Magnetic Anisotropy of Individual Nanomagnets Embedded in Biological Systems Determined by Axi-Asymmetric X-Ray Transmission Microscopy. ACS Nano.

[48] Marqués-Marchán J., Jaafar M., Ares P., Gubieda A.G., Berganza E., Abad A., Fdez-Gubieda M.L., Asenjo A. (2024). Magnetic Imaging of Individual Magnetosome Chains in Magnetotactic Bacteria. Biomater. Adv..

[49] Dunlap M.K., Ryan D.P., Goodwin P.M., Sheehan C.J., Werner J.H., Majumder S., Hollingsworth J.A., Gelfand M.P., Van Orden A. (2023). Nanoscale Imaging of Quantum Dot Dimers Using Time-Resolved Super-Resolution Microscopy Combined with Scanning Electron Microscopy. Nanotechnology.

[50] Goldstein J.I., Newbury D.E., Echlin P., Joy D.C., Lyman C.E., Lifshin E., Sawyer L., Michael J.R. (2003). Scanning Electron Microscopy and X-ray Microanalysis: Third Edition.

[51] Goldstein J.I., Newbury D.E., Echlin P., Joy D.C., Lyman C.E., Lifshin E., Sawyer L., Michael J.R., Goldstein J.I., Newbury D.E., Echlin P., Joy D.C., Lyman C.E., Lifshin E., Sawyer L., Michael J.R. (2003). Ambient-Temperature Specimen Preparation of Biological Material. Scanning Electron Microscopy and X-ray Microanalysis: Third Edition.

[52] Goldstein J.I., Newbury D.E., Echlin P., Joy D.C., Lyman C.E., Lifshin E., Sawyer L., Michael J.R., Goldstein J.I., Newbury D.E., Echlin P., Joy D.C., Lyman C.E., Lifshin E., Sawyer L., Michael J.R. (2003). Electron Beam–Specimen Interactions. Scanning Electron Microscopy and X-ray Microanalysis: Third Edition.

[53] Sacco M.A., Gualtieri S., Santos A., Mendes B., Raffaele R., Tarallo A.P., Verrina M.C., Ranno F., Monterossi M.D., Ricci P. (2025). Scanning Electron Microscopy Techniques in the Analysis of Gunshot Residues: A Literature Review. Appl. Sci..

[54] Abd Mutalib M., Rahman M.A., Othman M.H.D., Ismail A.F., Jaafar J., Hilal N., Ismail A.F., Matsuura T., Oatley-Radcliffe D. (2017). Chapter 9—Scanning Electron Microscopy (SEM) and Energy-Dispersive X-Ray (EDX) Spectroscopy. Membrane Characterization.

[55] Goldstein J.I., Newbury D.E., Echlin P., Joy D.C., Lyman C.E., Lifshin E., Sawyer L., Michael J.R., Goldstein J.I., Newbury D.E., Echlin P., Joy D.C., Lyman C.E., Lifshin E., Sawyer L., Michael J.R. (2003). Specimen Preparation of Hard Materials: Metals, Ceramics, Rocks, Minerals, Microelectronic and Packaged Devices, Particles, and Fibers. Scanning Electron Microscopy and X-ray Microanalysis: Third Edition.

[56] Bektas I., Yildirim N.B. (2025). Molecular Characterization of Bacterial Cellulose Producing Bacillus Strains Isolated From Soil. J. Basic. Microbiol..

[57] Oestreicher Z., Pérez-Guzmán L., Casillas-Ituarte N.N., Hostetler M.R., Mumper E., Bazylinski D.A., Lower S.K., Lower B.H. (2022). Thermophilic Magnetotactic Bacteria from Mickey Hot Springs, an Arsenic-Rich Hydrothermal System in Oregon. ACS Earth Space Chem..

[58] Schaible G.A., Kohtz A.J., Cliff J., Hatzenpichler R. (2022). Correlative SIP-FISH-Raman-SEM-NanoSIMS Links Identity, Morphology, Biochemistry, and Physiology of Environmental Microbes. ISME Commun..

[59] Muangkaew W., Thanomsridetchai N., Tangwattanachuleeporn M., Ampawong S., Sukphopetch P. (2024). Unveiling Lodderomyces Elongisporus as an Emerging Yeast Pathogen: A Holistic Approach to Microbiological Diagnostic Strategies. Mycopathologia.

[60] Kontomaris S.V., Stylianou A., Chliveros G., Malamou A. (2023). Overcoming Challenges and Limitations Regarding the Atomic Force Microscopy Imaging and Mechanical Characterization of Nanofibers. Fibers.

[61] Magazzù A., Marcuello C. (2023). Investigation of Soft Matter Nanomechanics by Atomic Force Microscopy and Optical Tweezers: A Comprehensive Review. Nanomaterials.

[62] Aziz A., Shaikh H., Abbas A., Zehra K.E., Javed B. (2024). Microscopic Techniques for Nanomaterials Characterization: A Concise Review. Microsc. Res. Tech..

[63] Bîrleanu C., Pustan M., Șerdean F., Merie V. (2021). AFM Nanotribomechanical Characterization of Thin Films for MEMS Applications. Micromachines.

[64] Joshi J., Homburg S.V., Ehrmann A. (2022). Atomic Force Microscopy (AFM) on Biopolymers and Hydrogels for Biotechnological Applications—Possibilities and Limits. Polymers.

[65] Parvej M.S., Wang X., Jiang L. (2020). AFM Based Nanomechanical Characterization of Cellulose Nanofibril. J. Compos. Mater..

[66] Xia F., Youcef-Toumi K. (2022). Review: Advanced Atomic Force Microscopy Modes for Biomedical Research. Biosensors.

[67] Duverger W., Tsaka G., Khodaparast L., Khodaparast L., Louros N., Rousseau F., Schymkowitz J. (2024). An End-to-End Approach for Single-Cell Infrared Absorption Spectroscopy of Bacterial Inclusion Bodies: From AFM-IR Measurement to Data Interpretation of Large Sample Sets. J. Nanobiotechnol.

[68] Gulati K., Adachi T. (2023). Profiling to Probing: Atomic Force Microscopy to Characterize Nano-Engineered Implants. Acta Biomater..

[69] Winkler R., Ciria M., Ahmad M., Plank H., Marcuello C. (2023). A Review of the Current State of Magnetic Force Microscopy to Unravel the Magnetic Properties of Nanomaterials Applied in Biological Systems and Future Directions for Quantum Technologies. Nanomaterials.

[70] Joshua A.M., Cheng G., Lau E.V. (2023). Soft Matter Analysis via Atomic Force Microscopy (AFM): A Review. Appl. Surf. Sci. Adv..

[71] Temiryazev A.G., Krayev A.V., Temiryazeva M.P. (2021). Two Dynamic Modes to Streamline Challenging Atomic Force Microscopy Measurements. Beilstein J. Nanotechnol..

[72] Wang W., Zhang K., Zhang W., Hou Y., Chen Y. (2021). Multifunctional Cantilevers for Simultaneous Enhancement of Contact Resonance and Harmonic Atomic Force Microscopy. Nanotechnology.

[73] Xu X., Feng H., Zhao Y., Shi Y., Feng W., Loh X.J., Vancso G.J., Guo S. (2024). AFM-Based Nanomechanics and Machine Learning for Rapid and Non-Destructive Detection of Bacterial Viability. Cell Rep. Phys. Sci..

[74] Efremov Y.M., Suter D.M., Timashev P.S., Raman A. (2022). 3D Nanomechanical Mapping of Subcellular and Sub-Nuclear Structures of Living Cells by Multi-Harmonic AFM with Long-Tip Microcantilevers. Sci. Rep..

[75] Shahina Z., Bhat S.V., Ndlovu E., Sultana T., Körnig A., Dague É., Dahms T.E.S., Gupta V.K., Tuohy M. (2022). Cellulomics of Live Yeast by Advanced and Correlative Microscopy. Laboratory Protocols in Fungal Biology: Current Methods in Fungal Biology.

[76] Marcuello C. (2022). Present and Future Opportunities in the Use of Atomic Force Microscopy to Address the Physico-Chemical Properties of Aquatic Ecosystems at the Nanoscale Level. Int. Aquat. Res..

[77] Lefèvre C.T., Bernadac A., Yu-Zhang K., Pradel N., Wu L.-F. (2009). Isolation and Characterization of a Magnetotactic Bacterial Culture from the Mediterranean Sea. Environ. Microbiol..

[78] Frankel R.B., Bazylinski D.A., Johnson M.S., Taylor B.L. (1997). Magneto-Aerotaxis in Marine Coccoid Bacteria. Biophys. J..

[79] Oestreicher Z., Valverde-Tercedor C., Chen L., Jimenez-Lopez C., Bazylinski D.A., Casillas-Ituarte N.N., Lower S.K., Lower B.H. (2012). Magnetosomes and Magnetite Crystals Produced by Magnetotactic Bacteria as Resolved by Atomic Force Microscopy and Transmission Electron Microscopy. Micron.

[80] Zahn C., Keller S., Toro-Nahuelpan M., Dorscht P., Gross W., Laumann M., Gekle S., Zimmermann W., Schüler D., Kress H. (2017). Measurement of the Magnetic Moment of Single Magnetospirillum Gryphiswaldense Cells by Magnetic Tweezers. Sci. Rep..

[81] Šoltýs J., Feilhauer J., Vetrova I., Tóbik J., Bublikov K., Ščepka T., Fedor J., Dérer J., Cambel V. (2020). Magnetic-Field Imaging Using Vortex-Core MFM Tip. Appl. Phys. Lett..

[82] Stiufiuc G.F., Stiufiuc R.I. (2024). Magnetic Nanoparticles: Synthesis, Characterization, and Their Use in Biomedical Field. Appl. Sci..

[83] Shimoshige H., Nakajima Y., Kobayashi H., Yanagisawa K., Nagaoka Y., Shimamura S., Mizuki T., Inoue A., Maekawa T. (2017). Formation of Core-Shell Nanoparticles Composed of Magnetite and Samarium Oxide in Magnetospirillum Magneticum Strain RSS-1. PLoS ONE.

[84] Li W., Yu L., Zhou P., Wang G., Xu B., Cheng Z., Xu W. (2014). Properties of Magnetite Nanoparticles Produced by Magnetotactic Bacteria. J. Wuhan. Univ. Technol.-Mat. Sci. Edit..

[85] Shi Y., Wang L., Li L., Feng C., Cao Y. (2025). Innovative Progress of LSPR-Based Dark-Field Scattering Spectral Imaging in the Biomedical Assay at the Single-Particle Level. ChemistryOpen.

[86] Fathollahi Arani S., Zeinoddini M., Saeedinia A.R., Danesh N.M., Robatjazi S.M. (2024). LSPR-Based Colorimetric Aptasensor Design for Rapid and Simple Detection of Vibrio Cholerae O1. Appl. Biochem. Microbiol..

[87] Tang L., Casas J., Venkataramasubramani M. (2013). Magnetic Nanoparticle Mediated Enhancement of Localized Surface Plasmon Resonance for Ultrasensitive Bioanalytical Assay in Human Blood Plasma. Anal. Chem..

[88] Mostufa S., Rezaei B., Azizi E., Wang Y.A., Li C., Gómez-Pastora J., He R., Wu K. (2025). Characterizing the Physicochemical Properties of Magnetic Nanoparticles by a Surface Plasmon Resonance Approach. AIP Adv..

[89] Han X.X., Rodriguez R.S., Haynes C.L., Ozaki Y., Zhao B. (2022). Surface-Enhanced Raman Spectroscopy. Nat. Rev. Methods Primers.

[90] Wang C., Meloni M.M., Wu X., Zhuo M., He T., Wang J., Wang C., Dong P. (2019). Magnetic Plasmonic Particles for SERS-Based Bacteria Sensing: A Review. AIP Adv..

[91] Cheng S., Tu Z., Zheng S., Khan A., Yang P., Shen H., Gu B. (2024). Development of a Magnetically-Assisted SERS Biosensor for Rapid Bacterial Detection. Int. J. Nanomed..

[92] Yadav V.K., Pramanik S., Alghamdi S., Atwah B., Qusty N.F., Babalghith A.O., Solanki V.S., Agarwal N., Gupta N., Niazi P. (2025). Therapeutic Innovations in Nanomedicine: Exploring the Potential of Magnetotactic Bacteria and Bacterial Magnetosomes. Int. J. Nanomed..

[93] Mobed A., Darvishi M., Kohansal F., Dehfooli F.M., Alipourfard I., Tahavvori A., Ghazi F. (2024). Biosensors; Nanomaterial-Based Methods in Diagnosing of *Mycobacterium tuberculosis*. J. Clin. Tuberc. Other Mycobact. Dis..

[94] Sun Z., Ji X., Lu S., Du J. (2025). Shining a Light on Environmental Science: Recent Advances in SERS Technology for Rapid Detection of Persistent Toxic Substances. J. Environ. Sci..

[95] Rostami B., Nansen C. Application of Active Acoustic Transducers in Monitoring and Assessment of Terrestrial Ecosystem Health—A Review—Rostami—2022—Methods in Ecology and Evolution—Wiley Online Library. https://besjournals.onlinelibrary.wiley.com/doi/full/10.1111/2041-210X.14004.

[96] Viggen E.M., Arnestad H.K. (2023). Modelling Acoustic Radiation from Vibrating Surfaces around Coincidence: Radiation into Fluids. J. Sound Vib..

[97] Gouda M., Ghazzawy H.S., Alqahtani N., Li X. (2023). The Recent Development of Acoustic Sensors as Effective Chemical Detecting Tools for Biological Cells and Their Bioactivities. Molecules.

[98] Borodina I.A., Zaitsev B.D., Alsowaidi A.K.M., Karavaeva O.A., Guliy O.I. (2022). A Biological Sensor Based on the Acoustic Slot Mode Using Microbial Cells for the Determination of Ampicillin. Acoust. Phys..

[99] Nair M.P., Teo A.J.T., Li K.H.H. (2022). Acoustic Biosensors and Microfluidic Devices in the Decennium: Principles and Applications. Micromachines.

[100] Minakov A.V., Pryazhnikov M.I., Damdinov B.B., Nemtsev I.V. (2022). Acoustic Spectroscopy Study of the Bulk Viscosity of Nanosuspensions. Acoust. Phys..

[101] Kulkarni M.B., Ayachit N.H., Aminabhavi T.M. (2022). Biosensors and Microfluidic Biosensors: From Fabrication to Application. Biosensors.

[102] Guliy O.I., Zaitsev B.D., Semyonov A.P., Alsowaidi A.K.M., Teplykh A.A., Karavaeva O.A., Borodina I.A. (2022). Microbial Acoustic Sensor Test-System Based on a Piezoelectric Resonator with a Lateral Electric Field for Kanamycin Detection in Liquid. Ultrasonics.

[103] Durukan Y., Shevelko M., Peregudov A., Popkova E., Shevchenko S. (2020). The Effect of a Rotating Medium on Bulk Acoustic Wave Polarization: From Theoretical Considerations to Perspective Angular Motion Sensor Design. Sensors.

[104] Länge K. (2022). Bulk and Surface Acoustic Wave Biosensors for Milk Analysis. Biosensors.

[105] Thakur A., Kumar A. (2023). Recent Trends in Nanostructured Carbon-Based Electrochemical Sensors for the Detection and Remediation of Persistent Toxic Substances in Real-Time Analysis. Mater. Res. Express.

[106] Mousaabadi K.Z., Ensafi A.A., Rezaei B. (2022). Simultaneous Determination of Some Opioid Drugs Using Cu-Hemin MOF@MWCNTs as an Electrochemical Sensor. Chemosphere.

[107] Christ-Ribeiro A., Maciel J.V., Bier E.M., Pinto J.S., Dias D. (2022). Application of Electrochemical Sensors in the Determination of Synthetic Dyes in Foods or Beverages and Their Toxicological Effects on Human Health: A Review. Food Anal. Methods.

[108] Xing L., Zhang W., Fu L., Lorenzo J.M., Hao Y. (2022). Fabrication and Application of Electrochemical Sensor for Analyzing Hydrogen Peroxide in Food System and Biological Samples. Food Chem..

[109] Hulanicki A., Glab S., Ingman F. (1991). Chemical Sensors: Definitions and Classification. Pure Appl. Chem..

[110] Baranwal J., Barse B., Gatto G., Broncova G., Kumar A. (2022). Electrochemical Sensors and Their Applications: A Review. Chemosensors.

[111] Barhoum A., Hamimed S., Slimi H., Othmani A., Abdel-Haleem F.M., Bechelany M. (2023). Modern Designs of Electrochemical Sensor Platforms for Environmental Analyses: Principles, Nanofabrication Opportunities, and Challenges. Trends Environ. Anal. Chem..

[112] Bhattacharyya A.S. (2024). Conducting Polymers in Biosensing: A Review. Chem. Phys. Impact.

[113] He Q., Wang B., Liang J., Liu J., Liang B., Li G., Long Y., Zhang G., Liu H. (2023). Research on the construction of portable electrochemical sensors for environmental compounds quality monitoring. Mater. Today Adv..

[114] Maheshwaran S., Chen W.-H., Lin S.-L., Ghorbani M., Hoang A.T. (2024). Metal Oxide-Based Electrochemical Sensors for Pesticide Detection in Water and Food Samples: A Review. Environ. Sci. Adv..

[115] Lee J., Kim M.C., Soltis I., Lee S.H., Yeo W.-H. (2023). Advances in Electrochemical Sensors for Detecting Analytes in Biofluids. Adv. Sens. Res..

[116] Lin T., Xu Y., Zhao A., He W., Xiao F. (2022). Flexible Electrochemical Sensors Integrated with Nanomaterials for *in Situ* Determination of Small Molecules in Biological Samples: A Review. Anal. Chim. Acta.

[117] Zhou H., Ma X., Sailjoi A., Zou Y., Lin X., Yan F., Su B., Liu J. (2022). Vertical Silica Nanochannels Supported by Nanocarbon Composite for Simultaneous Detection of Serotonin and Melatonin in Biological Fluids. Sens. Actuators B Chem..

[118] Singh R., Gupta R., Bansal D., Bhateria R., Sharma M. (2024). A Review on Recent Trends and Future Developments in Electrochemical Sensing. ACS Omega.

[119] Boonkaew S., Jang I., Noviana E., Siangproh W., Chailapakul O., Henry C.S. (2021). Electrochemical Paper-Based Analytical Device for Multiplexed, Point-of-Care Detection of Cardiovascular Disease Biomarkers. Sens. Actuators B Chem..

[120] Crapnell R.D., Ferrari A.G.-M., Dempsey N.C., Banks C.E. (2022). Electroanalytical Overview: Screen-Printed Electrochemical Sensing Platforms for the Detection of Vital Cardiac, Cancer and Inflammatory Biomarkers. Sens. Diagn..

[121] Fessler M., Su Q., Jensen M.M., Zhang Y. (2023). Electroactivity of the Magnetotactic Bacteria Magnetospirillum Magneticum AMB-1 and Magnetospirillum Gryphiswaldense MSR-1. Front. Environ. Sci. Eng..

[122] Nakhlband A., Kholafazad-Kordasht H., Rahimi M., Mokhtarzadeh A., Soleymani J. (2022). Applications of Magnetic Materials in the Fabrication of Microfluidic-Based Sensing Systems: Recent Advances. Microchem. J..

[123] Revathy R., Sajini T., Augustine C., Joseph N. (2023). Iron-Based Magnetic Nanomaterials: Sustainable Approaches of Synthesis and Applications. Results Eng..

[124] Jeyakumar P., Debnath C., Vijayaraghavan R., Muthuraj M. (2023). Trends in Bioremediation of Heavy Metal Contaminations. Environ. Eng. Res..

[125] Adampourezare M., Hasanzadeh M., Hoseinpourefeizi M.-A., Seidi F. (2023). Iron/Iron Oxide-Based Magneto-Electrochemical Sensors/Biosensors for Ensuring Food Safety: Recent Progress and Challenges in Environmental Protection. RSC Adv..

[126] Wu F., Liu J. (2022). Decorated Bacteria and the Application in Drug Delivery. Adv. Drug Deliv. Rev..

[127] Chen C., Wang P., Chen H., Wang X., Halgamuge M.N., Chen C., Song T. (2022). Smart Magnetotactic Bacteria Enable the Inhibition of Neuroblastoma under an Alternating Magnetic Field. ACS Appl. Mater. Interfaces.

[128] Shen Y., Tissot A., Serre C. (2022). Recent Progress on MOF-Based Optical Sensors for VOC Sensing. Chem. Sci..

[129] Anwar S., Ali G., Maab H., Khan Q., Akhtar S., Karim S., Khan M., Maqbool M. (2022). Six Band Terahertz Absorption in Metamaterial for Designing Optical Filters, and Sensors. Opt. Quant. Electron..

[130] Khan S.A., Khan N.Z., Xie Y., Abbas M.T., Rauf M., Mehmood I., Runowski M., Agathopoulos S., Zhu J. (2022). Optical Sensing by Metamaterials and Metasurfaces: From Physics to Biomolecule Detection. Adv. Opt. Mater..

[131] Tang J., Qiu G., Wang J. (2022). Recent Development of Optofluidics for Imaging and Sensing Applications. Chemosensors.

[132] Pendão C., Silva I. (2022). Optical Fiber Sensors and Sensing Networks: Overview of the Main Principles and Applications. Sensors.

[133] Davison N.B., Gaffney C.J., Kerns J.G., Zhuang Q.D. (2022). Recent Progress and Perspectives on Non-Invasive Glucose Sensors. Diabetology.

[134] Karim K., Lamaoui A., Amine A. (2023). Paper-Based Optical Sensors Paired with Smartphones for Biomedical Analysis. J. Pharm. Biomed. Anal..

[135] Lv J., Wang J., Yang L., Liu W., Fu H., Chu P.K., Liu C. (2024). Recent Advances of Optical Fiber Biosensors Based on Surface Plasmon Resonance: Sensing Principles, Structures, and Prospects. Sens. Diagn..

[136] Park J.-H., Cho Y.-W., Kim T.-H. (2022). Recent Advances in Surface Plasmon Resonance Sensors for Sensitive Optical Detection of Pathogens. Biosensors.

[137] Philip A., Kumar A.R. (2022). The Performance Enhancement of Surface Plasmon Resonance Optical Sensors Using Nanomaterials: A Review. Coord. Chem. Rev..

[138] Uniyal A., Srivastava G., Pal A., Taya S., Muduli A. (2023). Recent Advances in Optical Biosensors for Sensing Applications: A Review. Plasmonics.

[139] Qin J., Jiang S., Wang Z., Cheng X., Li B., Shi Y., Tsai D.P., Liu A.Q., Huang W., Zhu W. (2022). Metasurface Micro/Nano-Optical Sensors: Principles and Applications. ACS Nano.

[140] Amjad A., Xian X. (2025). Optical Sensors for Transdermal Biomarker Detection: A Review. Biosens. Bioelectron..

[141] Nan M., Darmawan B.A., Go G., Zheng S., Lee J., Kim S., Lee T., Choi E., Park J.-O., Bang D. (2023). Wearable Localized Surface Plasmon Resonance-Based Biosensor with Highly Sensitive and Direct Detection of Cortisol in Human Sweat. Biosensors.

[142] Hair M.E., Gerkman R., Mathis A.I., Halámková L., Halámek J. (2019). Noninvasive Concept for Optical Ethanol Sensing on the Skin Surface with Camera-Based Quantification. Anal. Chem..

[143] Sultana N., Dewey H., Budhathoki-Uprety J. (2022). Optical Detection of pH Changes in Artificial Sweat Using Near-Infrared Fluorescent Nanomaterials. Sens. Diagn..

[144] Sharifi A.R., Ardalan S., Tabatabaee R.S., Soleimani Gorgani S., Yousefi H., Omidfar K., Kiani M.A., Dincer C., Naghdi T., Golmohammadi H. (2023). Smart Wearable Nanopaper Patch for Continuous Multiplexed Optical Monitoring of Sweat Parameters. Anal. Chem..

[145] Jaishi L.R., Yu J., Ding W., Tsow F., Xian X. (2024). A Novel Colorimetric Tuning Fork Sensor for Ammonia Monitoring. Sens. Actuators B Chem..

[146] Cui Y., Cao Q., Zheng X., Dong H., Wang J., Xu F., Qian L., Wang D. (2024). A Wearable Bracelet for Simultaneous Monitoring of Transcutaneous Carbon Dioxide and Pulse Rates. Adv. Electron. Mater..

[147] Tipparaju V.V., Mora S.J., Yu J., Tsow F., Xian X. (2021). Wearable Transcutaneous CO_2_ Monitor Based on Miniaturized Nondispersive Infrared Sensor. IEEE Sens. J..

[148] Kaur B., Kumar S., Kaushik B.K. (2023). Novel Wearable Optical Sensors for Vital Health Monitoring Systems—A Review. Biosensors.

[149] Li C., Tang Q., Wei H., Liu J., Wang Q., Wang Y., Du Z., Wang J., Xu R., Bi Y. (2022). Smart Wearable Fluorescence Sensing of Bacterial Pathogens and Toxic Contaminants by Eu^3+^-Induced Sodium Alginate/Ag Nanoparticle Aggregates. ACS Appl. Nano Mater..

[150] Pandey P.S., Raghuwanshi S.K., Shadab A., Ansari M.T.I., Tiwari U.K., Kumar S. (2022). SPR Based Biosensing Chip for COVID-19 Diagnosis—A Review. IEEE Sens. J..

[151] Chen H., Li Y., Wang Y., Ning P., Shen Y., Wei X., Feng Q., Liu Y., Li Z., Xu C. (2022). An Engineered Bacteria-Hybrid Microrobot with the Magnetothermal Bioswitch for Remotely Collective Perception and Imaging-Guided Cancer Treatment. ACS Nano.

[152] Garcés V., González A., Gálvez N., Delgado-López J.M., Calvino J.J., Trasobares S., Fernández-Afonso Y., Gutiérrez L., Dominguez-Vera J.M. (2022). Magneto-Optical Hyperthermia Agents Based on Probiotic Bacteria Loaded with Magnetic and Gold Nanoparticles. Nanoscale.

[153] Dudchenko N., Pawar S., Perelshtein I., Fixler D. (2022). Magnetite Nanoparticles: Synthesis and Applications in Optics and Nanophotonics. Materials.

[154] Maurya P., Mukherjee M.D., Ranjan K.R., Pratap Singh R., Adetunji C.O., Singh R.L., Singh J., Solanki P.R., Singh K.R.B. (2023). Chapter 3—Role of Nanobiotechnology in Maintaining a Hygienic Environment for the Livestock. Nanobiotechnology for the Livestock Industry.

[155] Inês A., Cosme F. (2025). Biosensors for Detecting Food Contaminants—An Overview. Processes.

[156] Clark L.C., Lyons C. (1962). Electrode Systems for Continuous Monitoring in Cardiovascular Surgery. Ann. N. Y. Acad. Sci..

[157] Katey B., Voiculescu I., Penkova A.N., Untaroiu A. (2023). A Review of Biosensors and Their Applications. ASME Open J. Eng..

[158] Smutok O., Katz E. Biosensors: Electrochemical Devices—General Concepts and Performance. https://www.mdpi.com/2079-6374/13/1/44.

[159] Bhatia D., Paul S., Acharjee T., Ramachairy S.S. (2024). Biosensors and Their Widespread Impact on Human Health. Sens. Int..

[160] Naresh V., Lee N. (2021). A Review on Biosensors and Recent Development of Nanostructured Materials-Enabled Biosensors. Sensors.

[161] Manoharan Nair Sudha Kumari S., Thankappan Suryabai X. (2024). Sensing the Future─Frontiers in Biosensors: Exploring Classifications, Principles, and Recent Advances. ACS Omega.

[162] Rashidy Ahmady A., Khan S., Han H., Gao W., Hosseinidoust Z., Didar T.F. (2025). Micro- and Nano-Bots for Infection Control. Adv. Mater..

[163] Sannigrahi S., Kumar A.S., Mathiyarasu J., Suthindhiran K. (2023). Detection of *Escherichia coli* in Food Samples by Magnetosome-Based Biosensor. Biotechnol. Bioproc E.

[164] de Souza Freire L., Ruzo C.M., Salgado B.B., Gandarilla A.M.D., Romaguera-Barcelay Y., Tavares A.P.M., Sales M.G.F., Cordeiro I., Lalwani J.D.B., Matos R. (2022). An Electrochemical Immunosensor Based on Carboxylated Graphene/SPCE for IgG-SARS-CoV-2 Nucleocapsid Determination. Biosensors.

[165] Xia Q., Jiang H., Liu X., Yin L., Wang X. (2024). Advances in Engineered Nano-Biosensors for Bacteria Diagnosis and Multidrug Resistance Inhibition. Biosensors.

[166] Deng X., Su Y., Xu M., Gong D., Cai J., Akhter M., Chen K., Li S., Pan J., Gao C. (2023). Magnetic Micro/nanorobots for Biological Detection and Targeted Delivery. Biosens. Bioelectron..

[167] Chen H., Zhou T., Li S., Feng J., Li W., Li L., Zhou X., Wang M., Li F., Zhao X. (2023). Living Magnetotactic Microrobots Based on Bacteria with a Surface-Displayed CRISPR/Cas12a System for Penaeus Viruses Detection. ACS Appl. Mater. Interfaces.

[168] Zimina T.M., Sitkov N.O., Gareev K.G., Fedorov V., Grouzdev D., Koziaeva V., Gao H., Combs S.E., Shevtsov M. (2022). Biosensors and Drug Delivery in Oncotheranostics Using Inorganic Synthetic and Biogenic Magnetic Nanoparticles. Biosensors.

[169] Gotovtsev P. (2023). Microbial Cells as a Microrobots: From Drug Delivery to Advanced Biosensors. Biosensors.

[170] Chen C.-Y., Chen C.-F., Yi Y., Chen L.-J., Wu L.-F., Song T. (2014). Construction of a Microrobot System Using Magnetotactic Bacteria for the Separation of Staphylococcus Aureus. Biomed. Microdevices.

[171] Chen C., Chen L., Yi Y., Chen C., Wu L.-F., Song T. (2016). Killing of Staphylococcus Aureus via Magnetic Hyperthermia Mediated by Magnetotactic Bacteria. Appl. Env. Microbiol..

[172] Chen C., Chen L., Wang P., Wu L.-F., Song T. (2017). Magnetically-Induced Elimination of *Staphylococcus Aureus* by Magnetotactic Bacteria under a Swing Magnetic Field. Nanomed. Nanotechnol. Biol. Med..

[173] Song S.-J., Mayorga-Martinez C.C., Vyskočil J., Častorálová M., Ruml T., Pumera M. (2023). Precisely Navigated Biobot Swarms of Bacteria Magnetospirillum Magneticum for Water Decontamination. ACS Appl. Mater. Interfaces.

[174] Kotakadi S.M., Borelli D.P.R., Nannepaga J.S. (2022). Therapeutic Applications of Magnetotactic Bacteria and Magnetosomes: A Review Emphasizing on the Cancer Treatment. Front. Bioeng. Biotechnol..

[175] Gupta J., Hashmi A.A. (2024). Magnetotactic Bacteria-Synthesized Nanoparticles and Their Applications. Green Synthesis of Nanomaterials.

[176] Smid P., Shcherbakov V., Petersen N. (2015). Microscopic Observation of Magnetic Bacteria in the Magnetic Field of a Rotating Permanent Magnet. Rev. Sci. Instrum..

[177] Bidaud C.C., Monteil C.L., Menguy N., Busigny V., Jézéquel D., Viollier É., Travert C., Skouri-Panet F., Benzerara K., Lefevre C.T. (2022). Biogeochemical Niche of Magnetotactic Cocci Capable of Sequestering Large Polyphosphate Inclusions in the Anoxic Layer of the Lake Pavin Water Column. Front. Microbiol..

[178] Dubay M.M., Acres J., Riekeles M., Nadeau J.L. (2023). Recent Advances in Experimental Design and Data Analysis to Characterize Prokaryotic Motility. J. Microbiol. Methods.

[179] Potrč T., Kralj S., Nemec S., Kocbek P., Kreft M.E. (2023). The Shape Anisotropy of Magnetic Nanoparticles: An Approach to Cell-Type Selective and Enhanced Internalization. Nanoscale.

[180] Taveira I., Cypriano J., Guimarães J., Vieira L.C.G., Junior J.F.G., Enrich-Prast A., Abreu F. (2025). Ecology and Spatial Distribution of Magnetotactic Bacteria in Araguaia River Floodplain. Environ. Microbiol. Rep..

[181] Hamilton S., Regan D., Payne L., Langbein W., Borri P. (2022). Sizing Individual Dielectric Nanoparticles with Quantitative Differential Interference Contrast Microscopy. Analyst.

[182] Wang X., Wang H., Wang J., Liu X., Hao H., Tan Y.S., Zhang Y., Zhang H., Ding X., Zhao W. (2023). Single-Shot Isotropic Differential Interference Contrast Microscopy. Nat. Commun..

[183] Hodoroaba V.-D., Hodoroaba V.-D., Unger W.E.S., Shard A.G. (2020). Chapter 4.4—Energy-Dispersive X-Ray Spectroscopy (EDS). Characterization of Nanoparticles.

[184] Makela A.V., Schott M.A., Madsen C.S., Greeson E.M., Contag C.H. (2022). Magnetic Particle Imaging of Magnetotactic Bacteria as Living Contrast Agents Is Improved by Altering Magnetosome Arrangement. Nano Lett..

[185] Xu Y., Zhang Y., Li C., Ye Z., Bell S.E.J. (2023). SERS as a Probe of Surface Chemistry Enabled by Surface-Accessible Plasmonic Nanomaterials. Acc. Chem. Res..

[186] Mandal P., Tewari B.S. (2022). Progress in Surface Enhanced Raman Scattering Molecular Sensing: A Review. Surf. Interfaces.

[187] Zhou S., Li J., Zhang Q., Tong Y., Qi X., Duan Y., Zhang X., Luo Z., Li Y. (2024). Recent Advance on Fiber Optic SPR/LSPR-Based Ultra-Sensitive Biosensors Using Novel Structures and Emerging Signal Amplification Strategies. Opt. Laser Technol..

[188] Comanescu C. (2023). Recent Advances in Surface Functionalization of Magnetic Nanoparticles. Coatings.

[189] Kassem A., Abbas L., Coutinho O., Opara S., Najaf H., Kasperek D., Pokhrel K., Li X., Tiquia-Arashiro S. (2023). Applications of Fourier Transform-Infrared Spectroscopy in Microbial Cell Biology and Environmental Microbiology: Advances, Challenges, and Future Perspectives. Front. Microbiol..

[190] Nandiyanto A.B.D., Ragadhita R., Fiandini M. (2023). Interpretation of Fourier Transform Infrared Spectra (FTIR): A Practical Approach in the Polymer/Plastic Thermal Decomposition. Indones. J. Sci. Technol..

[191] Ketenoglu D. (2022). A General Overview and Comparative Interpretation on Element-Specific X-Ray Spectroscopy Techniques: XPS, XAS, and XRS. X-Ray Spectrom..

[192] Zhang T., Liu Y., Tong L., Yu J., Lin S., Li Y., Fan H.J. (2023). Oxidation State Engineering in Octahedral Ni by Anchored Sulfate to Boost Intrinsic Oxygen Evolution Activity. ACS Nano.

[193] Gandarias L., Jefremovas E.M., Gandia D., Marcano L., Martínez-Martínez V., Ramos-Cabrer P., Chevrier D.M., Valencia S., Fernández Barquín L., Fdez-Gubieda M.L. (2023). Incorporation of Tb and Gd Improves the Diagnostic Functionality of Magnetotactic Bacteria. Mater. Today Bio.

[194] Feng F., Li Q., Sun X., Yao L., Wang X. (2024). Tumor Microenvironment-Responsive Magnetotactic Bacteria-Based Multi-Drug Delivery Platform for MRI-Visualized Tumor Photothermal Chemodynamic Therapy. Biology.

